# The human bone marrow harbors a CD45^−^ CD11B^+^ cell progenitor permitting rapid microglia‐like cell derivative approaches

**DOI:** 10.1002/sctm.20-0127

**Published:** 2020-12-09

**Authors:** Andreas Bruzelius, Isabel Hidalgo, Antonio Boza‐Serrano, Anna‐Giorgia Hjelmér, Amelie Tison, Tomas Deierborg, Johan Bengzon, Tania Ramos‐Moreno

**Affiliations:** ^1^ Division of Neurosurgery, Department of Clinical Sciences Lund Skåne University Hospital, Lund Stem Cell Center Lund Sweden; ^2^ Department of Experimental Medical Science and Lund Stem Cell Center BMC Lund University Lund Sweden; ^3^ Institution for Laboratory Medicine, Division of Molecular Hematology, Faculty of Medicine Lund University Lund Sweden; ^4^ Departamento de Bioquimica y Biologia Molecular, Facultad de Farmacia e Instituto de Biomedicina de Sevilla (IBiS), Hospital Universitario Virgen del Rocio/CSIC/Universidad de Sevilla Seville Spain; ^5^ Department of Experimental Medical Science, Experimental Neuroinflammation Laboratory, Bio Medical Center (BMC). Lund University Lund Sweden

**Keywords:** bone marrow, common myeloid progenitor, human bone marrow, microglia, microglial precursor, microglia‐like cell in vitro model, primitive myeloid progenitor, pluripotent stem cell

## Abstract

Microglia, the immune sentinel of the central nervous system (CNS), are generated from yolk sac erythromyeloid progenitors that populate the developing CNS. Interestingly, a specific type of bone marrow‐derived monocyte is able to express a yolk sac microglial signature and populate CNS in disease. Here we have examined human bone marrow (hBM) in an attempt to identify novel cell sources for generating microglia‐like cells to use in cell‐based therapies and in vitro modeling. We demonstrate that hBM stroma harbors a progenitor cell that we name stromal microglial progenitor (STR‐MP). STR‐MP single‐cell gene analysis revealed the expression of the consensus genetic microglial signature and microglial‐specific genes present in development and CNS pathologies. STR‐MPs can be expanded and generate microglia‐like cells in vitro, which we name stromal microglia (STR‐M). STR‐M cells show phagocytic ability, classically activate, and survive and phagocyte in human brain tissue. Thus, our results reveal that hBM harbors a source of microglia‐like precursors that can be used in patient‐centered fast derivative approaches.

## Graphical abstract

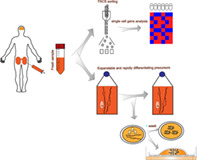

**Significance statement**

The authors believe that the discovery of an intermediate specified myeloid microglia progenitor will open the way for developing novel patient‐centered approaches and in vitro modeling. The use of stromal microglial progenitor as the starting point in derivative microglia‐like cell approaches, instead of a somatic cell to first generate human pluripotent stem cells, reduces the risk of teratoma formation and the overall waiting time of current microglia. Thus, these results will be of broad general interest to a number of high‐profile areas of research, including stem cells and in vitro modeling, neurodegeneration, neuropsychiatry, brain tumors, bone marrow stromal cells, hematopoietic system, and human ontogeny.

## 1 INTRODUCTION

Microglia are generated from yolk sac erythromyeloid progenitors (EMPs) that populate the developing central nervous system (CNS) to become its resident macrophage.^1^, ^2^ Microglia play a key role in the brain development, ensure maintenance and function during adulthood,^3^ and are involved in the onset and progression of CNS pathologies, including neurodegenerative and psychiatric disorders^4^ as well as malignant brain tumors.^5^ Microglia constitute a target for therapy.^6^ Development of drugs tries to target microglia for controlling immunomodulation of the CNS environment^7^ and also possesses a potential in cell therapy applications as microglia exhibit neuroprotection in ischemia^8^ and are scavengers for amyloid β (Aβ) in Alzheimer's disease.^9^ Presently, the potential therapeutic application of human microglia‐like cells is limited by the lack of CNS microglial sources and fast and safe stem cell approaches for deriving microglia. Current methods of generating induced pluripotent stem cells (iPSCs) that subsequently can be differentiated to microglia‐like cells are time‐consuming (≥4 months), have low efficiency, and are potentially associated with tumor risks when used for transplantation purposes.^10^, ^11^ The use of embryonic stem cells suffers also from ethical concerns and limited access,^12^ whereas approaches using microglia‐like cells derived from bone marrow monocytes bring functional differences that are associated to their unique roles in lesion formation in the curse of CNS degeneration.^13^, ^14^, ^15^ Interestingly, a specific type of murine bone marrow‐derived microglia‐like cells has been reported to be able to populate CNS in disease and to express a yolk sac microglial signature.^16^ In the present work, we have examined human bone marrow (hBM) in an attempt to identify the presence of a cell source for generating human microglia‐like cells in fast and reliable derivative approaches.

We demonstrate the presence of a nonhematopoietic cell population, herein named stromal microglial progenitors (STR‐MPs), in the stromal compartment of the hBM. Single‐cell gene analysis shows that this cell population shares a microglial genetic signature, can be culture‐expanded in mesenchymal stromal cell (MSC) cultures, and can generate microglia‐like cells (stromal microglia [STR‐M]) in vitro. The STR‐M retain microglial markers in vitro, have phagocytic and immune polarization abilities and microglia‐like cell morphology, and survive in an ex vivo human brain organotypic model. We thus provide an efficient, quick, and nondisruptive procedure to generate human functional STR‐M, with the capacity to become polarized upon lipopolysaccharide (LPS) exposure and to phagocyte latex beads and Aβ fibrils, for use in in vitro microglial modeling and/or cell‐based therapies.

## 2 MATERIALS AND METHODS

### 2.1 Procurement of hBM stromal cells

Bone marrow was aspirated from the iliac crest of healthy donors (60 mL, n = 5, median age 24 years, range 21‐35 years). All participants in the study gave a written informed consent. The procedure was approved by the Swedish local ethics committee in Lund (Regionala Etikprövningsnämden Lund [EPN], protocol Dnr2009/12), and all experimental protocols were performed according to EPN's guidelines. Bone marrow mononuclear cells were isolated by density gradient centrifugation (LSM 1077 Lymphocyte; PAA Laboratories, Thermo Fisher Scientific, Waltham, Massachusetts) similar to that previously described,^17^, ^18^ but no RosetteSep Human Mesenchymal Stem Cell Enrichment Cocktail was performed. PharmLyse was used to further eliminate red blood cells from the cell preparation. Stromal cells were allowed to attach to plastic and were regularly culture‐expanded in MSC expansion medium (StemMACS MSC Expansion Media; Miltenyi Biotec, Bergisch Gladbach, Germany) supplemented with the broad‐spectrum antibiotic Primocin (1:1000; InvivoGen, San Diego, California).

### 2.2 Flow cytometry and cell sorting of STR‐M‐like cells

Single‐cell suspensions from the freshly isolated hBM of the five healthy donors (three female donors and two male donors aged between 21 and 35 years) were stained in fluorescence‐activated cell sorting (FACS) buffer (phosphate‐buffered saline [PBS; Gibco, Thermo Fisher Scientific] + 10% fetal bovine serum [Gibco] + 0.2 mM ethylenediamine tetraacetic acid [EDTA; Sigma‐Aldrich, St. Louis, Missouri]) for 30 minutes at 4°C with the following antibodies: Brilliant Violet 421‐cluster of differentiation (CD) 73 (AD2) (1/200) and Alexa Fluor 700‐CD90 (5E10) (1/200), both from Nordic BioSite, Täby, Sweden; phycoerythrin (PE)‐CD105 (266) (1/200), PE‐cyanine (Cy) 5‐human leukocyte antigen‐ABC isotype (HLA‐ABC) (G46‐2.6) (1/200), PE‐Cy7‐CD45 (H130) (1/200), Brilliant Violet 605‐CD11b (1/200), fluorescein isothiocyanate (FITC)‐CD14 (M5E2) (1/200), FITC‐CD19 (HIB19) (1/200), FITC‐CD34 (581) (1/200), and FITC‐HLA‐DR isotype (HLA‐DR) (G46‐6) (all from BD Biosciences, San Jose, California). Propidium iodide (P3566; Life Technologies, Thermo Fisher Scientific) at a concentration of 1/1000 in FACS buffer was used as a viability marker prior to acquisition. All cells were analyzed and sorted on a FACSAria IIu cell sorter (BD Biosciences).

### 2.3 RNA sequencing extraction and single‐cell gene analysis

Single HLA‐DR^−^ CD14^−^ CD19^−^ CD34^−^ CD45^−^ CD11b^+^ cells were sorted into 4 μL lysis buffer.^19^ Preamplification was performed using TaqMan primers and Taq/SSIII reaction mix (Invitrogen, Thermo Fisher Scientific). Linearity control and negative controls were included in each plate. Preamplification was performed according to a published polymerase chain reaction (PCR) protocol^19^ with an extended 50°C cycle. Complementary DNA was added to 48.48 Dynamic Array Chips (Fluidigm, South San Francisco, California) with individual TaqMan assays and quantitative PCR, analyzed on BioMarkHD (Fluidigm).

### 2.4 Data analysis

Quantitative PCR data were analyzed in the BiomarkHD analysis software (quality threshold 0.60, automatic global cycle threshold, and linear derivative baseline correction). After excluding controls, data were preprocessed in SCExV^20^ where cycle threshold values were inverted, normalized to median expression of each cell, and *z*‐score normalized. Unsupervised clustering analysis was performed using random forest clustering^21^ and principal component analysis, and correlations were measured using the Spearman rank method.

### 2.5 Cell culture of hBM MSCs and nonhematopoietic CD11b^+^ cells

Procured hBM MSCs were expanded with StemMACS MSC Expansion Media (MiltenyBiotec) supplemented with Primocin (1:1000, InvivoGen), which we refer to as expansion or basal medium (BM) interchangeably, in T75 plastic flasks (Thermo Fisher Scientific). When they reached confluence, cells were trypsinized using trypsin‐ (0.25%), phenol red (Thermo Fisher Scientific).

### 2.6 hBM MSCs and nonhematopoietic CD11b^+^ cell expansion in serum‐containing and serum‐free conditions

*For serum‐containing experiments*: When they reached ~90% confluence in T75 plastic flasks (Thermo Fisher Scientific), cells were kept in their StemMACS MSC Expansion Media (MiltenyBiotec) supplemented with Primocin (1:1000, InvivoGen) for 6 days, mimicking the serum‐free medium experiments (described in the paragraph below). Next, preconditioned cells were seeded in 24 multiwells onto laminin (Sigma‐Aldrich) and polyornithine (Advanced BioMatrix, Carlsbad, California) coated coverslips at a density of 80 000 to 100 000 cells per well. Next day after the seeding, cells were exposed to (a) only this expansion medium, BM; (b) BM supplemented with brain‐derived neurotrophic factor (BDNF) and human neutrophin 3 (NT3) (both from PeproTech, Rocky Hill, NJ; final concentration of 10 ng/mL) added fresh every second day for as long as required by the time points (2 or 4 weeks; Figure S1B); or (c) BM supplemented with macrophage colony‐stimulating factor (M‐CSF; 25 ng/mL; ThermoFisher Scientific), interleukin (IL)‐34 (100 ng/mL; PeproTech), and transforming growth factor (TGF) β‐1 (50 ng/mL; Militenyi) added fresh every second day for as long as required by the time points (2 or 4 weeks; Figure S1B).

*For serum‐free medium experiments*: Experimental protocol started when cells reached ~90% confluence in the T75 flasks; cells were then exposed to base medium consisting of Dulbecco's modified Eagle's medium/F12 (Gibco), 1× N2 (Gibco), 1× Non‐essential amino acids (NEAA;Gibco), and 1× B27 (Gibco), and supplemented with Primocin (1:1000, InvivoGen) for 6 days. Next, these preconditioned cells were seeded in 24 multiwells with coated coverslips at a density of 80 000 to 100 000 cells per well and treated as before, using the serum‐free medium, which we refer to as neuronal medium (NM), supplemented with growth factors or cytokines (CKs) for 2 or 4 weeks (Figure S1B).

### 2.7 Immunocytochemistry

After fixing with an ice‐cold paraformaldehyde 4% solution diluted in a 0.1 M potassium phosphate‐buffered solution (4% paraformaldehyde [PFA]) for 30 minutes at room temperature, cells were permeabilized with 0.25% Triton X‐100 for 15 minutes at room temperature. The cells were then blocked using donkey serum (1:20) for 30 minutes at room temperature before incubating with primary antibody for 2 hours and then with secondary donkey antibody for 1 hour coupled to either Cy3, Alexa Fluor 488, or Alexa Fluor 647 for anti‐chicken, goat, mouse, or rabbit IgG antibodies (all from Jackson ImmunoResearch Laboratories, West Grove, Pennsylvania; 1:200). Between each step the cells were washed with PBS three times for 5 minutes. Primary antibodies used were against the human CD11b (Abcam, Cambridge, Massachusetts; 1:200; rabbit), human ionized calcium‐binding adapter molecule 1 (Iba1; Abcam; 1:200; goat), human transmembrane protein 119 (TMEM119; Abcam; 1:200; rabbit), fractalkine or chemokine ligand 1 (CX3CR1; Abcam; 1:200; mouse), human CD33 (Abcam; 1:200; mouse), major histocompatibility class II HLA‐DR (Abcam; 1:200; mouse), and green fluorescent protein (GFP; Abcam; 1:200; chicken). As negative control the primary antibody was omitted. All antibodies used in the present work are listed in Table S6A.

### 2.8 Counting

Quantification analysis was done in an automated image analysis using the software Thermo Scientific HCS Studio: Cellomics Scan, version 6.6. A manual counting was added to exclude possible artifacts. The threshold levels were set to an internal control that contained only secondary antibodies. The high intensity levels were calculated per well as equal to or higher than the mean for the intensities plus twice the SD.

Quantification of cells derived from hBM MSCs was done manually after taking images at ×20 magnification using the filter for 4′,6‐diamidino‐2‐phenylindole U‐MNUA2 (excitation filter 365/5), tetramethylrhodamine/Cy3 49 005 (excitation filter band pass 454/15), and Cy5 49 009 (excitation filter band pass 640/15) under the epifluorescence microscope (Olympus, Tokyo, Japan) and using the software CellSens. A total of 10 visual fields per coverslip was used for cell counting, and a total of 50 to 100 cells per coverslip were counted for every condition using the ImageJ software. The high intensity levels were calculated by manually comparing the intensities between cells per field.

### 2.9 Microglial phagocytosis assay

Fluorescence latex beads of 1 μm diameter (Sigma‐Aldrich) were first preopsinized for 1 hour at 37°C at a ratio of 1:5 with fetal bovine serum (Gibco). Preopsinized beads were subsequently diluted in the different serum‐containing and serum‐free culture conditions for a final concentration of latex beads of 0.01% (Figure S2A). Cells were in contact with medium and preopsonized latex beads for 1 hour at 37°C and subsequently fixed with cold 4% PFA solution for a posterior immunocytochemistry study for CD11b and Iba1 expression.

### 2.10 Aβ oligomer preparation and treatment

Aβ (M1‐42) fluorescent peptides (wavelength 555) were prepared by diluting Aβ dimethylsulfoxide stock at a final 186 μM in PBS by incubating it at 37°C for 24 hours. This stock was brought to a final concentration of 0.5 μM in NM supplemented with CKs (NM + CK) or NM supplemented with neutrophins (NM + NT) and left over night (O/N) at 4°C to allow the formation of fibrils and oligomers before completely changing the medium of the cells. Presorted nonhematopoietic CD11b^+^ cells were seeded in a 96 multiwell, at a density of 50 000 cells per well, and, after being exposed to either NM + CK or NM + NT for 6 days, media containing the Aβ 42 oligomers and cells were incubated for 1 hour at 37°C, respectively, and fixed in cold 4% PFA for posterior analysis.

### 2.11 LPS induced activation

Presorted nonhematopoietic CD11b^+^ cells were seeded at a density of 80 to 100 × 10^3^ cells per well in 24 multiwell plates and treated as depicted in the scheme (Figure S1B). Cell cultures were incubated for 24 hours with 100 ng/mL of LPS from *Escherichia coli* O111:B4 (Sigma‐Aldrich) added to the different serum‐free culture conditions (Figure S2B). After 24‐hour incubation, supernatants were collected, frozen in dry ice, and stored at −80°C for a subsequent Meso Scale analysis.

### 2.12 Meso Scale

The release of proinflammatory CKs in culture media upon LPS activation was measured using Meso Scale (Meso Scale Diagnostics, Rockville, Maryland) plates with the proinflammatory panels for interferon‐γ, IL‐1β, IL‐2, IL‐4, IL‐5, IL‐6, IL‐8, IL‐10, IL‐12, and tumor necrosis factor‐α. The plates were developed using the 4× reading buffer diluted to a factor of 1× with distilled water, and the plates were read using the QuickPlex Q120 reader from Meso Scale. The detection ranges of the different CKs measured were as follows: IL‐1β (1670‐0.408 pg/mL), IL‐4 (1660‐0.405 pg/mL), IL‐12 (32200‐7.86 pg/mL), IL‐10 (3410‐0.833 pg/mL), interferon‐γ (938‐0.229 pg/mL), IL‐2 (2630‐0.642 pg/mL), IL‐5 (967‐0.236 pg/mL), IL‐6 (5720‐1.40 pg/mL), KC/GRO (1980‐0.483 pg/mL), and tumor necrosis factor‐α (627‐0.153 pg/mL).

### 2.13 Lentiviral transduction and antibiotic selection of human stromal cells

hBM MSCs along with the nonhematopoietic CD11b^+^ cells were transduced at passages 4 and 5 in T75 flasks at 80% confluence. Medium was completely changed with 10 mL of the StemMACS MSC Expansion Media (Milteny Biotec) containing 10 μL lentiviral construct (1.7 × 10^8^) carrying a noninducible construct for GFP with puromycin resistance (73 582; Addgene, Watertown, Massachusetts). Preparations of lentiviral particles were produced according to protocol from^22^ in a biosafety level 2 environment by the Vector Core Facility at the Lund Stem Cell Center. Next day after lentiviral transduction, medium was changed back to StemMACS MSC Expansion Media (Milteny Biotec) supplemented with Primocin (1:1000, InvivoGen). Forty‐eight hours after infection, cultures were treated with puromycin at a final concentration of 10 μg/mL for 5 days.

### 2.14 Coculture of human nonhematopoietic CD11b^+^ cells, stromal cells, and adult human cortex organotypic slice cultures

Adult human cortical tissue from three patients with glioma was obtained by informed consent from the patients according to guidelines approved by the Lund‐Malmö Ethical Committee. All three patients were male and aged 52 to 66 years, and tumors were located in the frontotemporal region in these cases. Tumor pathology varied: isocitrate dehydrogenase (IDH) wild‐type primary glioblastoma, IDH wild‐type secondary glioblastoma, and IDH‐mutated diffuse astrocytoma. In all cases, tissue for culturing was collected from magnetic resonance imaging (MRI)‐positive, macroscopically pathological parts of the tumor front.

The surgically resected tissue was placed in ice‐cold solution of artificial cerebrospinal fluid solution (119 mM NaCl, 26 mM NaHCO_3_, 2.5 mM KCl, 1.25 mM NaH_2_PO_4_, 1.3 mM MgSO_4_, and 11 mM glucose dissolved in Milli‐Q and bubbled with carbogen for 15 minutes before adding 2.5 mM CaCl_2_) and transferred to the slicing chamber of a vibratome (VT1200S; Leica, Wetzlar, Germany) where 250‐μm thick tissue slices were cut. The sliced tissue was transferred to prepared six‐well plates with equilibrated culture medium and cultured at 37°C in a humidified atmosphere of 5% CO_2_. Organotypic tissue slices were cultured on Alvetex Strata six‐well membrane inserts (AMS Biotechnology, Milton, UK) that had been presoaked in organotypic culture medium (neurobasal, 2% B27 without vitamin A, 0.5% Glutamax, and 10 μg/mL gentamicin; all from Thermo Fisher Scientific) in six‐well plates, for a total of 3 weeks, and every second day the inserts were transferred to a new plate with 6 mL fresh and equilibrated medium in similar fashion as previously reported by Miskinyte et al.^23^

At day 7, one well per medium was selected and grafted with GFP^+^ human predifferentiated nonhematopoietic CD11b^+^ as part of the hBM MSC cultures. Briefly, 10 μL of a suspension mix (1 000 000 GFP^+^ hBM MSCs) was collected into a cold glass capillary and injected as small drops stabbing the semidry slices at various sites in a randomized fashion trying to cover most of the surface. Slices were transplanted with 10 μL of suspension leading to approximately 300 000 cells per slice. Additional medium was added 30 minutes later to fully immerse the organotypic culture. The medium was changed every other day during the week, and coculture was maintained for 3 weeks before fixation. Brain tissue was fixed in 4% PFA solution, washed three times with PBS, and submerged in sucrose at an increase of 10% each day for 3 days, with the final day being 30%, and subsequently stored in antifreeze solution (30% ethylene glycol and 30% glycerol, both from VWR International, West Chester, Pennsylvania) in 0.012 M NaH_2_PO_4_·H_2_O and 0.031 M Na_2_HPO_4_·2H_2_O (both from Sigma‐Aldrich) at −20°C (Figure S2C).

### 2.15 iDISCO

Immunolabeling‐enabled three‐dimensional imaging of solvent‐cleared organs (iDISCO) was performed using the readily available protocol at https://idisco.info/idisco-protocol/ with slight modifications. In brief, samples were fixed with 4% PFA and prepared using the methanol pretreatment; samples were then incubated in permeabilization solution for 1 day followed by blocking solution for 1 day. Samples were incubated with the primary antibodies anti GFP (Abcam; 1:200; chicken), human CD11b (Abcam; 1:200; mouse), and TMEM119 (Abcam; 1:200; rabbit) and with secondary antibodies Cy3, Alexa Fluor 488, or Alexa Fluor 647 for anti‐chicken, goat, mouse, or rabbit IgG antibodies (all from Jackson ImmunoResearch Laboratories; 1:200); incubation was set to 2 days with washing steps between for 1 day. Succeeding clearing steps were done following the recommended times in the protocol. Samples where then mounted with PO‐PRO‐1 Iodide (Invitrogen) mixed with DABCO and imaged using a Zeiss LSM 780 confocal microscope (Carl Zeiss Microscopy GmbH, Jena, Germany).

### 2.16 Functional assays in the cocultures and histology

For the functional assays in the cocultures, adult human cortical tissue from one patient with glioma was obtained, sliced, and brought on the inserts as described in the previous section. Three to 5 days after slicing, a total of 30 000 STR‐MP cells per slice previously sorted were labeled with the fluorescent vital dye CellTracker Red CMTPX (Thermo Fisher Scientific) according to the manufacturer's instructions and were grafted the same day in similar fashion as described above. Cocultures were kept for 6 days in NM + CK (prepared as described above), and medium was changed every 3 days to a previously equilibrated NM + CK. At this time point, latex beads were added into NM + CK with LPS from *E. coli* O111:B4 (Sigma‐Aldrich) at a 100 ng/mL concentration, and the media of the cocultures were changed to this one. After 24 hours, cocultures were fixed in cold 4% PFA for 20 minutes and left in PBS for further histological analysis. Previous to image acquisition, cocultures were treated O/N at 4°C with a 1% Triton X‐100 in PBS permeabilizing solution containing PO‐PRO‐1 Iodide (1:200, Invitrogen).

### 2.17 Statistical analysis

All statistical analysis was performed using Graph Pad Prism version 7.0c. Data were assumed to fit a normal distribution, and statistical comparisons were made using unpaired, two‐tailed *t* tests for comparisons of two means between untreated groups and/or for comparisons between different time points within a condition; one‐tailed unpaired *t* test when comparing two groups exposed to a treatment, that is, LPS^24^; and, depending on the number of variables, one‐ or two‐way analysis of variance (ANOVA) followed by Tukey's test when comparing multiple groups. A value of *P* < .05 was considered statistically significant. Data are presented as means ± SEM.

## 3 RESULTS

### 3.1 Identifying STR‐MP cells

**FIGURE 1:**
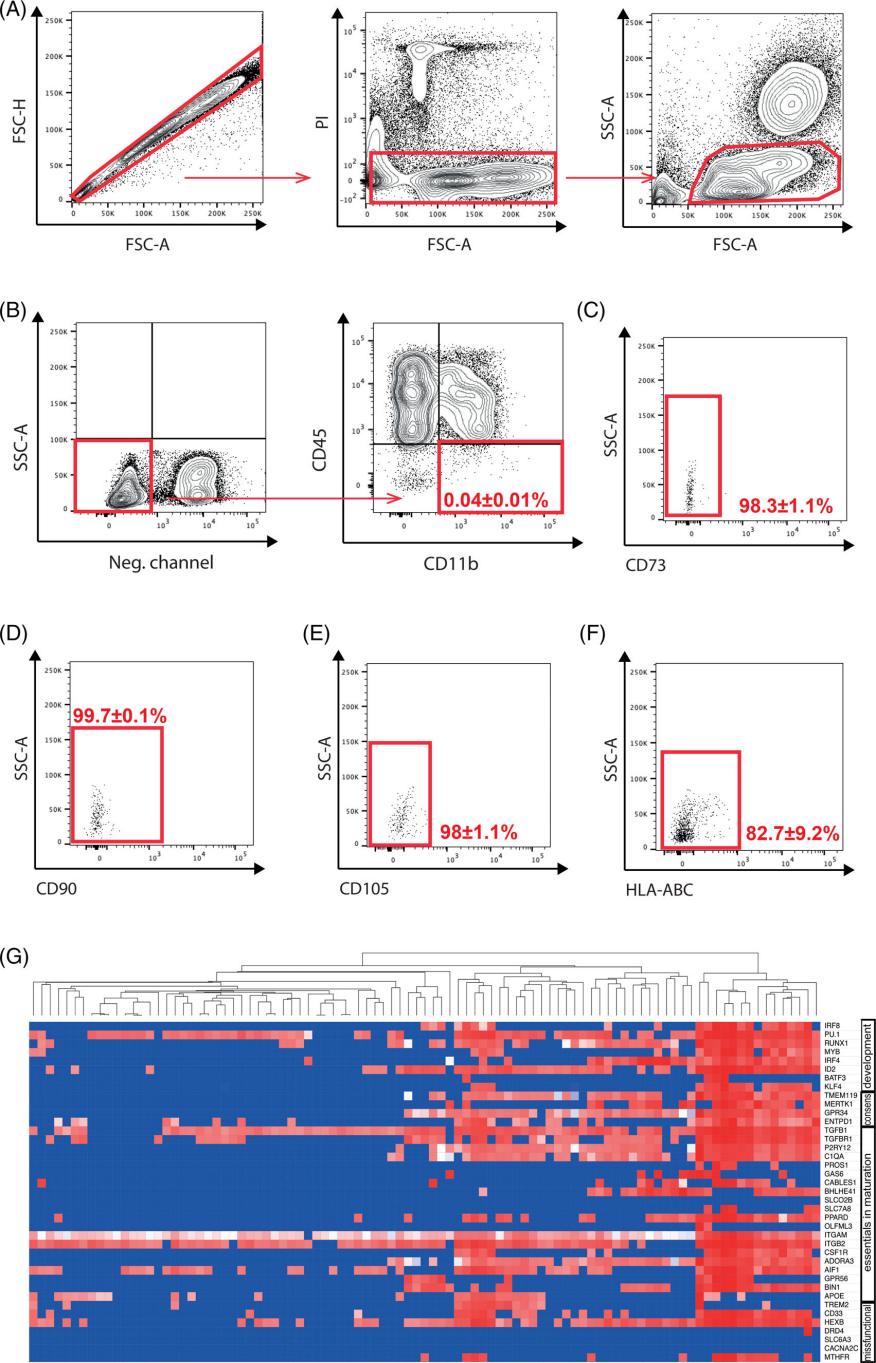
Human bone marrow stromal cells harbor nonhematopoietic CD11b^+^ cells that express microglial‐specific genes. A‐F, Representative case showing the gating strategy for detecting nonhematopoietic CD11b^+^ cells. Cells negative for markers HLA‐DR, CD14, CD19, and CD34 that label endothelial and hematopoietic lineages and hematopoietic stem cells were selected within the compartment of living cells. Selected cells (HLA‐DR^−^ CD14^−^ CD19^−^ CD34^−^/CD45^−^ CD11b^+^) were analyzed for CD90, CD73, CD105, and HLA‐ABC mesenchymal markers (n = 5 donors). G, Single‐cell gene analysis in the CD45^−^ CD11b^+^ nonhematopoietic nonendothelial compartment reveals a microglial expression pattern. Analyzed genes include the microglial consensus signature, genes involved in microglial development and maturation, and genes reported to be misfunctional in neurodegenerative and psychiatric disorders (n = 4 donors). Each column represents the gene analysis for a sorted cell, whereas the *y*‐axis provides a list of the genes. CD, cluster of differentiation; FSC‐A, forward scatter‐area; FSC‐H, forward scatter‐height; HLA‐ABC, human leukocyte antigen‐ABC isotype; PI, propidium iodide; SSC‐A, side scatter‐area

We hypothesized that, because the adult hematopoietic system derives from the hematopoietic stem cells originated during the definitive hematopoiesis,^25^, ^26^ if EMPs or EMP‐like cells are present in the adult hBM, they are likely to be part of the stromal fraction. In order to target the stromal fraction and avoid genetic drift, selection bias, and enrichment of specific populations in the samples, fresh isolated hBM was analyzed using flow cytometry (Figure 1A), initially to negatively select monocyte/macrophages. Within the remaining fraction, CD14, CD19, CD34, and HLA‐DR cell surface markers were used to remove hematopoietic and endothelial cells in the bone marrow extractions (Figure 1B). The absence of the above‐mentioned markers together with CD45 was used to help to identify the hBM stromal compartment^27^ from which CD11b^+^ cells were selected, with HLA‐DR^−^ CD14^−^ CD19^−^ CD34^−^ CD45^−^ CD11b^+^ cells constituting 0.04% ± 0.01% of hBM cells (n = 5; Figure 1B). Of the STR‐MP cells, 97.9% ± 1.1% were negative for CD73 (Figure 1C), 99.8% ± 0.1% were negative for CD90 (Figure 1D), 97.7% ± 1.1% were negative for CD105 (Figure 1E), and 82.7% ± 9.2% were negative for HLA‐ABC (Figure 1F), indicating the presence of an expected CD45^−^ CD11b^+^ human microglia‐like/myeloid progenitor profile^28^, ^29^ in the stromal fraction that does not share the mesenchymal molecular signature.^27^

Sorted CD45^−^ CD11b^+^ stromal cells were subjected to single‐cell analysis for essential, disease‐involved, and developmental microglial‐specific genes, as well as for the consensus microglial signature. The sorted cells expressed the typical microglial progenitor gene expression profile (Figure 1G; Table S1), including the myeloid‐associated transcription factors Runt related transcription factor 1 (RUNX1), Spi‐1 proto‐oncogene (PU.1) and Colony stimulation factor 1 receptor (CSF1R), which are essential for regulation of microglial cell development,^1^, ^30^ and the microglia‐enriched protein TMEM119 and beta‐hexosaminidase subunit beta (HEXB), indicating an early commitment toward microglial fate.^31^, ^32^ The STR‐MP single‐cell gene profile showed a canonical microglial gene expression pattern, including *TREM2*, *P2RY12*, *CD33*, *GPR34*, *GPR56*, *C1Q*, *CABLES1*, *BHLHE41*, *TMEM119*, *TGFBR1*, *ENTPD1*, *ITGB2*, *ITGAM*, *AIF*, *IRF8*, *ADORA3*, and *PPARD*.^30^, ^31^, ^33^, ^34^, ^35^ Because the vast majority of the microglial core gene signature is shared between immature progenitors and more mature microglial cells,^36^ our data suggest that a myeloid/microglia‐like progenitor cell compartment is present in the hBM stromal compartment, which we refer to as the STR‐MP compartment.

### 3.2 Culturing STR‐MP cells

In order to study if STR‐MP cells could derive microglia‐like cells in vitro, we first tried to find the optimal culturing conditions for STR‐MP. Eight attempts using five different donors for isolating STR‐MP from hBM samples showed that, unless reaching a minimum density of 30 000 STR‐MP cells per well in a 96 multiwell‐plate (76.93 mm^2^), STR‐MPs soon become apoptotic or quiescent when in contact with plastic (data not shown), suggesting that STR‐MP cells could benefit from a supportive cell layer for survival, as STR‐MP cells' profile was detected upon passages in the stromal cultures (Figure S1A).

**FIGURE 2:**
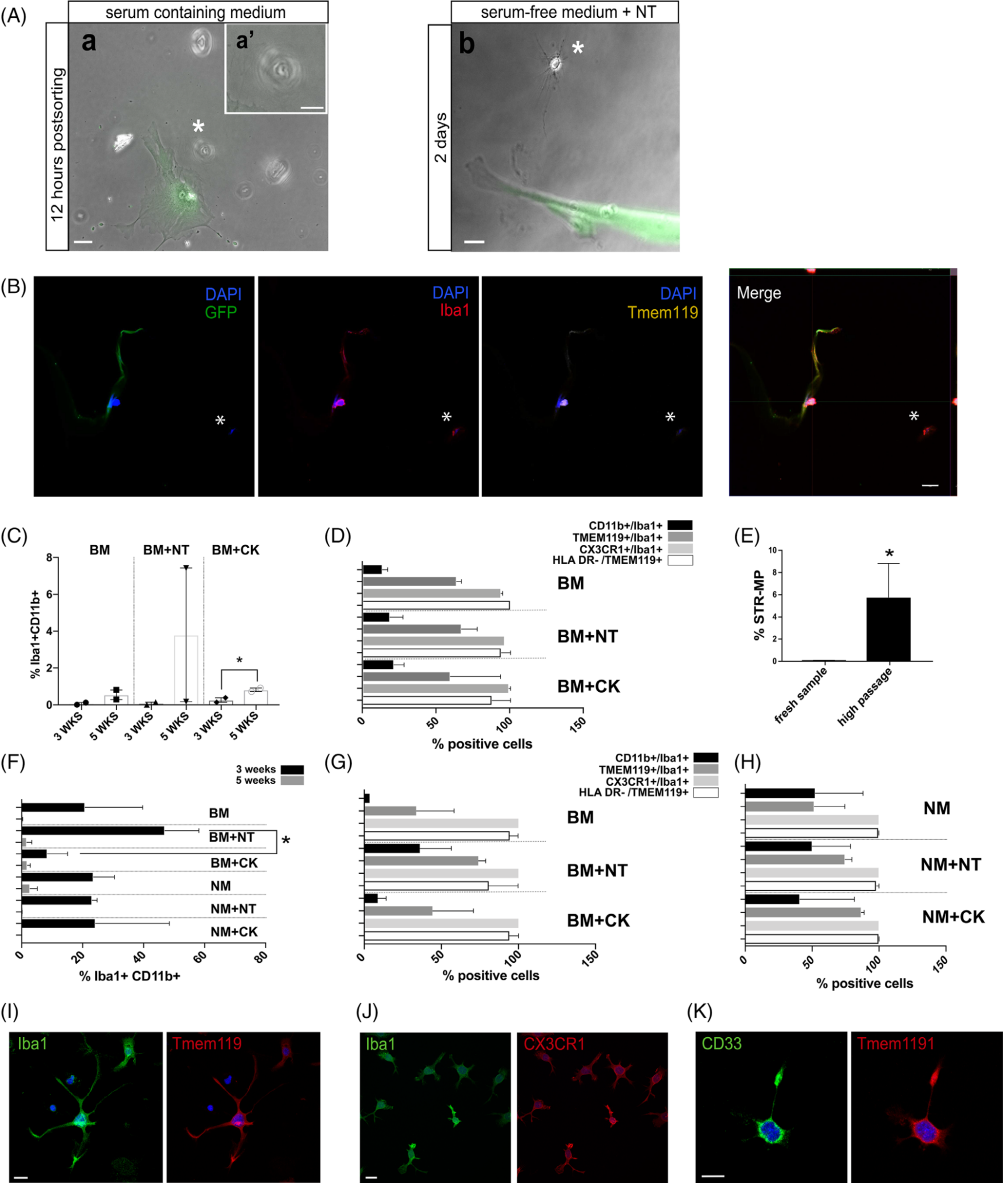
STR‐MP CD11b^+^ cells grow as part of mesenchymal stromal cultures, which act as a feeder cell layer. A, Twelve hours after sorting, sorted cells were seen attaching to plastic (a) showing a cubic cellular shape (denoted by an asterisk; scale bar, 20 μm; magnification in the upper frame to the right (a′); scale bar, 10 μm). In order to force a morphological change, cells were exposed to a serum‐free medium supplemented with neurotrophins to promote survival of human bone marrow‐derived mesenchymal stromal cells. After 2 days of serum‐free conditions, different ramified morphologies could be observed (b) (asterisk; scale bars, 20 μm; n = 2 donors, two repetitions). B, Cells were able to survive for 5 days on plastic and in contact with stromal cells. After 1 week of serum‐free condition exposure, cells were fixed in 4% and analyzed for Iba1 and TMEM119 expression. The arrow denotes a small GFP^−^ CD11b^+^ cell denoted by Hoechst and immunoreactive toward Iba1 and TMEM119. The asterisk shows a small GFP^−^ CD11b^+^ cell immunoreactive to Iba1 and negative to TMEM119 expression (scale bar, 20 μm; n = 2 donors, two repetitions). C, Analysis of the small nuclei compartment. The condition BM + CK showed a significant increase on Iba1 and CD11b immunoreactive cells (n = 2 donors; two‐way ANOVA; *P ≤ .05; **P ≤ .005; ***P ≤ .001). D, Expression of microglial markers Iba1, TMEM119, and CX3CR1 were included to determine if cells maintain their expression under the different culturing conditions. HLA‐DR was included as a negative control (Table S1). E, Fluorescence‐activated cell sorting analysis comparison of the STR‐MP compartment between high passage number (after passage 6) and fresh bone marrow sample shows that the STR‐MP compartment increases with passage number (0.04% ± 0.01% of STR‐MP in fresh sample vs 5.74% ± 3.05% of STR‐MP in cell cultures after passage 6, unpaired two‐tailed Student's *t* test, n = 5, *P* < .05). F, Stromal microglial CD11b^+^ cells were detected based on CD11b and Iba1 coexpression. The graph shows the percentage of these cells compared across the different medium conditions and two time points, 3 and 5 weeks. A higher percentage of Iba1 CD11b immunoreactive cells was observed at 3 weeks under the serum medium supplemented with neurotrophins (n = 2 donors; one‐way ANOVA; *P ≤ .05). G, The graph shows the percentage of cells in high passage cell cultures expressing the microglial markers Iba1, TMEM119, and CX3CR1 across culture serum‐containing conditions. HLA‐DR was included as a negative control (Table S2). H, The graph shows the percentage of cells in high passage cell cultures under serum‐free conditions for microglial markers Iba1, TMEM119, and CX3CR1. HLA‐DR was included as a negative control (Table S2). Note that the legend depicting the different immunocyochemistries applies for D, G, and H. Also, all cells were CX3CR1 immunoreactive. I, Representative Iba1 and TMEM119 cell in high passage cultures in the serum‐free supplemented with neurotrophins condition showing a microglial morphology at the 3‐week time point. Scale bar, 20 μm. J, Representative Iba1 and CX3CR1 cells in high passage cultures in the serum‐free supplemented with neurotrophins condition showing microglial morphologies at the 3‐week time point. Scale bar, 20 μm. K, Representative TMEM119 and CD33 cell in high passage cultures in the serum‐free supplemented with neurotrophins condition showing a microglial morphology at the 3‐week time point (counting can be found in Table S2; n = 2 donors; one‐way ANOVA; *P* > .05). Scale bar, 20 μm. All data presented as means ± SEM. BM, basal medium (expansion medium); BM + CK, basal medium supplemented with cytokines; BM + NT, basal medium supplemented with neutrophins; CD, cluster of differentiation; CX3CR1, fractalkine or chemokine ligand 1; DAPI, 4′,6‐diamidino‐2‐phenylindole; GFP, green fluorescent protein; HLA‐DR, human leukocyte antigen‐DR isotype; Iba1, ionized calcium‐binding adapter molecule 1; NM, neuronal medium; NM + CK, neuronal medium supplemented with cytokines; NM + NT, neuronal medium supplemented with neutrophins; NT, neutrophin; STR‐MP, stromal microglial progenitor; TMEM119 transmembrane protein 119

In a first attempt to follow and distinguish STR‐MP cells within the hBM stromal cultures, fresh hBM stromal samples were divided in two. Half of the samples were frozen, and the other half was expanded on plastic to be infected with a lentiviral vector coding for GFP followed by puromycin resistance on passage 4. One day after infection, cells were subjected to a subsequent 5‐day puromycin selection. Next, the frozen fresh bone marrow stromal cells corresponding to the same hBM samples were used to sort STR‐MP cells as described above, and the isolated STR‐MP cells were seeded with the corresponding donor, now being GFP^+^ and on passage 6 (Figures S1B and S2A). After 2 days, cells were transferred to serum‐free medium to facilitate macrophage‐like differentiation,^37^ medium that was supplemented with neurotrophins (NTs) to promote hBM MSC survival and, putatively, the STR‐M^38^ (Figure S1B). Cells were fixed at day 9 for an immunocytochemical analysis. GFP^−^ STR‐MP CD11b^+^ cells were detected both on plastic and in close contact with hBM stromal cells. Immunocharacterization of STR‐MP CD11b^+^ cells revealed high levels of Iba1 as well as TMEM119 expression (Figure 2B), indicating the maintenance of microglial marker previously detected on the single‐cell gene analysis (Figure 1G).

In order to find stimulatory conditions that can promote the presence of STR‐MP cells in vitro, nontransduced bone marrow stromal cell cultures at low passages (less than passage 6; based on Yang et al^39^) were subjected to different culturing conditions. The expansion medium containing serum was thus supplemented with either (a) NTs (human BDNF and NT3) or (b) CKs (IL‐34, M‐CSF, and TGF‐β).

The use of BDNF and NT3 (*the NT condition*) was expected to promote cell growth and survival of hBM MSCs and, putatively, the STR‐M.^38^, ^40^ The use of IL‐34, M‐CSF, and TGF‐β (*the CK condition*) was expected to facilitate culturing and differentiation into microglia‐like cells, as described when using derivative approaches from human stem pluripotent sources.^41^

Two time points of 3 and 5 weeks were included (Figure S1C) when cells were fixed for morphological and immunocytochemical studies of a combination of microglial markers as follows: Iba1 and CD11b, Iba1 and TMEM119, TMEM119 and HLA‐DR, and Iba1 and CX3CR1. Images were analyzed using the software ThermoScientific HCS Studio: Cellomics Scan, version 6.6 (see Section 2).

Two distinct populations defined by their Iba1 immunoreactivity and nuclear area size were observed under the different serum‐containing conditions (Figure S1D and Table S1A). Iba1^+^ CD11b^+^ cell nuclei area was restricted to 100 to 400 squared pixels (40‐156 μm^2^); thus, in subsequent analyses, we restricted counting to immunoreactive CD11b^+^ cells with a nucleus size equal to or less than 400 squared pixels (156 μm^2^). Within this group of small cells, CK conditions show an increase in the proportion of Iba1^+^ CD11b^+^ cells below 100 squared pixels in the stromal cell cultures with time (Figure 2C; 0.26% ± 0.12% at the 3‐week time point vs 0.81% ± 0.09% Iba1^+^ CD11b^+^ cells at the 5‐week time point in BM supplemented with CKs [BM + CK]; one‐tailed unpaired *t* test, *P* ≤ .05; n = 2 donors). Expression of microglial markers revealed that, on average and at 5 weeks under the same BM + CK condition, 59.16% ± 34.16% of the small cells are positive for TMEM119, 98.91% ± 1.08% are CX3CR1^+^, and 87.5% ± 12.5% do not express HLA‐DR. This pattern repeats across the different serum conditions, and the proportions do not vary between them, suggesting that this cell type and its expression pattern is maintained under all conditions (Figure 2D and Table S1B‐E).

A serum‐free medium condition was also included to closely mimic the CNS environment,^42^ facilitate macrophage‐like differentiation,^37^ and study a possible enhancement effect when supplementing with NTs or CKs. This study could not be completed as cells were not viable without serum.

Hence, the use of serum and a feeder cell layer such as the hBM stromal cells is highly recommended for an optimal expansion of the STR‐MP cells. The use of CK can promote the STR‐MP compartment by increasing their proportion in the stromal cultures upon time of exposure, and STR‐MP cultured cells retain the expression of microglial markers, that is, CD11b, Iba1, TMEM119, and CX3XR1.

### 3.3 Long‐term culturing of STR‐MP cells

The clonal complexity of MSCs vary upon cell expansion and is believed to depend on the selection of dominant MSC clones.^43^ In order to address if cell expansion affects the fate of STR‐MP so that the STR‐MP cell clone becomes dominant and its abundance in vitro is promoted upon passages, hBM stromal cultures were first expanded until apparent morphological changes due to passaging were observed (after passage 6, Figure S1E,F, based on Yang et al^39^). Cells were collected and stained for the cocktail of markers previously used for FACS and analysis in fresh bone marrow samples. The proportion of STR‐MP at high passages compared with the proportion of STR‐MP from fresh samples showed that in vitro cell expansion promotes the presence of STR‐MP cells in culture (0.04% ± 0.01% of STR‐MP in fresh sample vs 5.74% ± 3.05% of STR‐MP in cell cultures after passage 6, unpaired two‐tailed Student's *t* test, n = 5, *P* < .05; Figure 2E).

We next addressed the influence of in vitro long‐term culturing on the STR‐MP cellular characteristics, as functionality and differentiation of cultured cells can be affected and are important to consider for stem cell‐based therapies.^39^ Our results show that the use of NT in serum‐containing medium significantly increased Iba1^+^ CD11b^+^ cell numbers when compared with the CK‐supplemented medium at 3 weeks (Figure 2F and Table S2; 46.96 ± 11.15 BM supplemented with neutrophins vs 8.42 ± 6.63 BM + CK; unpaired, one‐tailed Student's *t* test, *P* ≤ .05; n = 2 donors; passage numbers 7‐8) but were significantly reduced to zero at 5 weeks in all conditions (Figure 2G and Table S2A‐D), and, similar to the low passaging cell cultures, STR‐MP high passaging cells defined by their expression on Iba1 and CD11b retain the expression of microglial markers (Figure 2G,H).

Long‐term cultured Iba1^+^ CD11b‐high cells were also able to survive under serum‐free condition (Figure 2H‐K and Table S2E‐H). In serum‐containing medium, the expression of TMEM119 can vary between 34.06% ± 24.31% and 74.15% ± 4.61% of Iba1^+^ cells, whereas in serum‐free medium the percentage of Iba1^+^ cells expressing TMEM119 varies between 51.52% ± 22.94% and 86.67% ± 2.21%. In serum‐free and serum‐containing conditions, all Iba1^+^ cells express CX3CR1 (Table S2G). Regarding the absence of HLA‐DR, between 80.85% ± 19.14% and 94.35% ± 5.6% of TMEM119^+^ cells do not express HLA‐DR in serum‐supplemented medium, and over 99.04% ± 0.96% lack HLA‐DR expression in serum‐free medium (Figure 2H and Table S2H). Thus, STR‐M defined as Iba1^+^ CD11b‐high cells in stromal cell cultures can survive in both serum‐containing and serum‐free conditions for up to 5 weeks. Moreover, STR‐MP cells significantly increase their numbers upon exposure of NT in a serum‐containing medium for 2 weeks and retain the expression of microglial markers but are not detected after this time.

**FIGURE 3:**
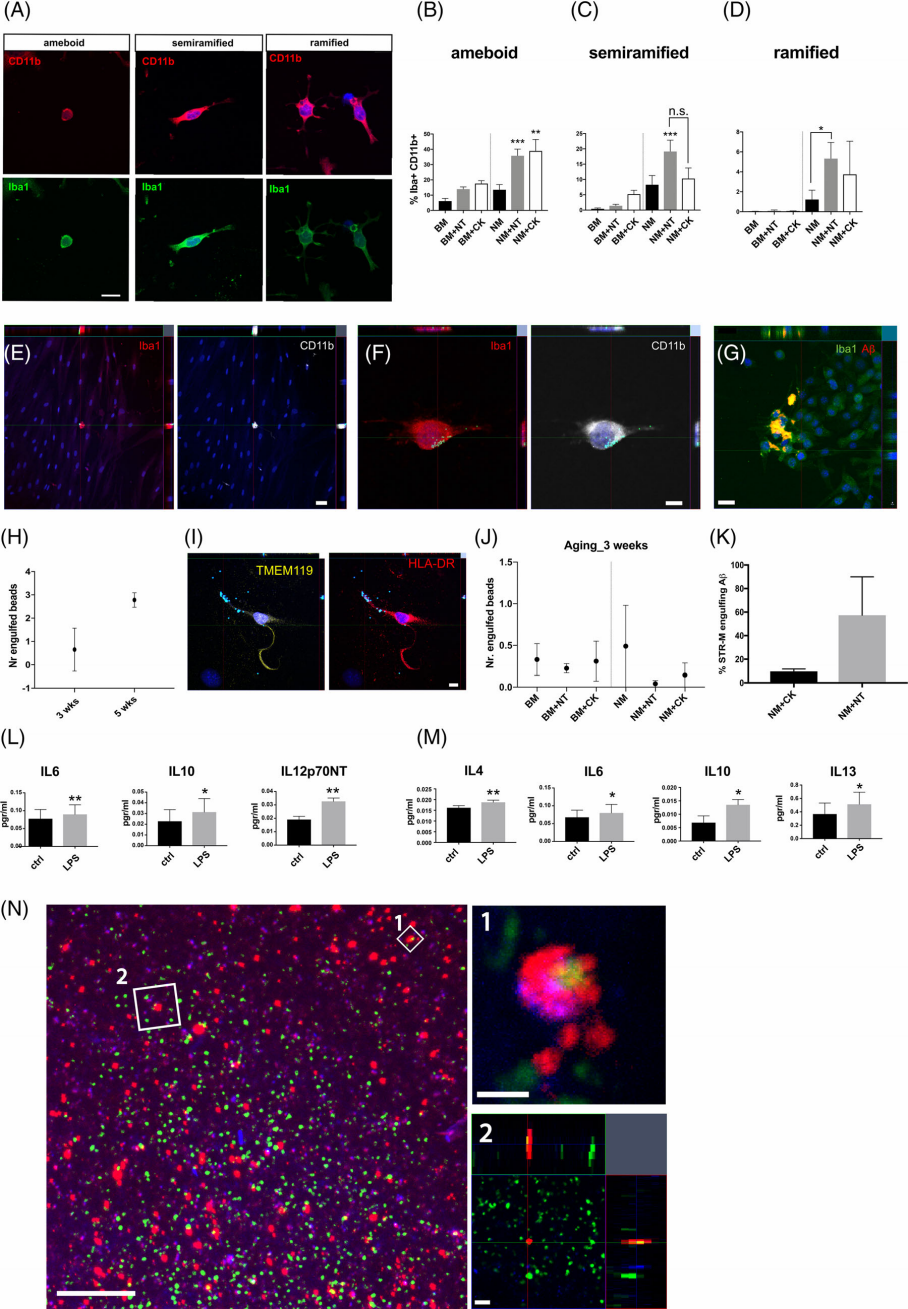
Characterization of high passage stromal microglial CD11b^+^ cells. A, Examples of ameboid, semiramified, and ramified morphologies typically observed in high passage cultures under serum‐free conditions, shown to be immune‐reactive to CD11b and Iba1. Scale bar, 20 μm. B, Graph showing the percentage of the ameboid morphology in serum‐free conditions (13.64% ± 3.25% in NM, 35.88% ± 4.21% in NM + NT, and 21.35 ± 4.89 in NM + CK; n = 2 donors; one‐way ANOVA; **P ≤ .005; ***P ≤ .001). C, Graph showing the percentage of semiramified morphology in serum‐free conditions (8.34% ± 2.93% in NM, 19.2% ± 3.63% in NM + NT, and 10.37% ± 3.37% in NM + CK; ***P ≤ .001). D, Graph showing the percentage of ramified morphology in serum‐free conditions. Note that in all conditions the serum‐free medium supplemented with neurotrophins showed higher percentages of the more complex morphologies (1.24% ± 0.92% in NM, 5.34% ± 1.6% in NM + NT, and 3.7 ± 3.31 in NM + CK; n = 2 donors; one‐way ANOVA; *P ≤ .05). E, Functional analysis of stromal microglial CD11b^+^ cells in low and high passage cultures. Representative image showing low passage cultures with an engulfing nonhematopoietic CD11b^+^ cell. Scale bar, 50 μm. F, Representative image showing high passage cultures with engulfing nonhematopoietic CD11b^+^ cells under the NM + CK condition. Scale bar, 10 μm. G, Representative orthogonal view of a confocal image showing presorted nonhematopoietic stromal microglial progenitor (STR‐MP) CD11b^+^ cells positive for Iba1 engulfing red fluorescent Aβ peptides at 6 days after being exposed to NM + CK condition. Green: Iba1 staining. Red: Aβ fibrils. Scale bar, 20 μm. H, Representative graph showing the number of engulfed beads per Iba1^+^ CD11b^+^ cell in the NM + CK condition at high passage upon time. No difference was found between the 3‐ and 5‐week time points (n = 2 donors; unpaired, two‐tailed Student's *t* test; *P > .05). I, Representative picture showing an HLA‐DR^+^ TMEM119^+^ cell engulfing latex beads. Scale bar, 10 μm. J, Graph showing the number of engulfed beads per HLA‐DR^+^ TMEM119^+^ cell under the different culture conditions at 3 weeks, as a representative example of the engulfment activity of HLA‐DR^+^ TMEM119^+^ cells (n = 2 donors; one‐way ANOVA; *P* > .05). K, Graph representing the percentage of sorted stromal microglial cells engulfing Aβ fibrils after being exposed to NM + NT and NM + CK conditions for 6 days (unpaired, two‐tailed Student's *t* test, *P* > .05; n = 3 repetitions). L, Classic activation of stromal microglia after 5 days of exposure to neutrophins in serum‐free conditions. An increase of the proinflammatory cytokine secretion of IL‐6, IL‐10, and IL‐12p70NT was detected upon 24‐hour exposure to LPS (n = 3 donors; paired, one‐tailed Student's *t* test, *P < .05; **P < .01). M, Classic activation of stromal microglia after 5 days of exposure to CK in serum‐free conditions. An increased secretion of the proinflammatory cytokines secretion of IL‐4, IL‐6, IL‐10, and IL‐13 was detected (n = 3 donors; paired, one‐tailed Student's *t* test, *P < .05; **P < .01). N, Representative image of sorted STR‐MP cells at 6‐day coculture in a low‐grade human glioblastoma resection. STR‐MPs were previously labeled with the fluorescent vital dye CellTracker Red CMTPX. Their derivatives are present at the edges and at the center of the resected slice and interact with and phagocytose green fluorescent protein‐positive (GFP^+^) latex beads. Scale bar, 100 μm. (1) One μm thick confocal magnification picture showing a transplanted cell engulfing GFP^+^ latex beads. Scale bar, 20 μm. (2) Orthogonal view showing phagocytosed GFP^+^ latex beads in a STR‐MP derivative (red). Scale bar, 10 μm. All data presented as means ± SEM. Aβ, amyloid β; BM, basal medium (expansion medium); BM + CK, basal medium supplemented with cytokines; BM + NT, basal medium supplemented with neutrophins; CD, cluster of differentiation; ctrl, control; HLA‐DR, human leukocyte antigen‐DR isotype; Iba1, ionized calcium‐binding adapter molecule 1; IL, interleukin; LPS, lipopolysaccharide; n.s., not significant; NM, neuronal medium; NM + CK, neuronal medium supplemented with cytokines; NM + NT, neuronal medium supplemented with neutrophins; TMEM119, transmembrane protein 119

Classic microglial morphologies were observed under serum‐free conditions in high passage cell cultures. Cells could be classified into three main morphological types (based on Au and Ma^44^): (a) *round‐shaped*, indicative of either a pluripotent cell or an activated‐like microglial state; (b) *semiramified*, when cells show one extension sprouting from its soma and indicative of a possible resting‐like status; and (c) *ramified*, when the cell shows more than one extension sprouting from its soma and also indicative of a resting‐like status (Figure 3A). A blinded manual analysis, where the culture condition being analyzed was not known by the researcher, was performed to determine morphologies under the different serum‐free conditions, revealing that the serum‐free condition supplemented with NT promotes semiramified and ramified morphologies in Iba1^+^ CD11b‐high cells, whereas the CK serum‐free condition promotes ameboid cell morphologies (Figure 3B‐D and Table S3; ordinary one‐way ANOVA, *P* ≤ .05; n = 2 donors; passage number 7‐8). Thus, the use of NT in a serum‐free medium promotes the morphological maturation of the STR‐Ms in high passage cultures.

### 3.4 Phagocytosis and classic activation in CD11b^+^ cells

To further demonstrate the microglial nature of Iba1^+^ CD11b^+^ cells, we assessed them, in low and high passage cultures, for phagocytosis with latex beads and Aβ fibrils and cell polarization, classic microglial characteristics. Iba1^+^ CD11b^+^ cells showed phagocytosis abilities in both low and high passage cultures under all conditions (Figure 3E‐K and Tables S4A,B and S5A,B; unpaired, one‐tailed Student's *t* test, *P* ≥ .05; n = 2 donors). No significant increase in the number of phagocytosed GFP^+^ latex beads per Iba1^+^ CD11b^+^ cell was detected in high passage cultures between time points (Figure 3H and Table S5A,B; unpaired, two‐tailed Student's *t* test, *P* ≤ .05; n = 2 donors). The small percentage of TMEM119^+^ and HLA‐DR^+^ cells present in the cultures could phagocyte beads (Figure 3I,J and Table S5A,B; unpaired, two‐tailed Student's *t* test, *P* > .05; n = 2 donors) and may be an indirect indication of the antigen‐presenting nature of these cells.

To determine if CD11b^+^ cells can be “classically activated,” cultures were exposed to bacterial LPS. In order to work with a pure population of STR‐Ms, hBM MSCs were expanded, and STR‐MPs were sorted and seeded in 96 multiwell plates at a confluence of 30 000 cells per well. The experimental design was restricted to the different serum‐free conditions in high passage cultures, taking advantage of the higher STR‐M content and avoiding the presence of serum, which can alter the experimental outcome.^45^ Our results show that, after being exposed to NT and CK conditions for only 5 days, STR‐Ms can be classically activated (Figure 3L,M). In the case of being exposed to NT, the proinflammatory CKs IL‐6 (0.077 ± 0.025 control vs 0.089 ± 0.026 upon LPS exposure), IL‐10 (0.022 ± 0.010 control vs 0.031 ± 0.012 upon LPS exposure), and IL‐12p70NT (0.01 ± 0.002 control vs 0.03 ± 0.002 upon LPS exposure) are detected in the supernatant after 24‐hour exposure to LPS (n = 3 donors; Figure 3L), whereas IL‐4 (0.016 ± 0.001 control vs 0.018 ± 0.001 upon LPS exposure), IL‐6 (0.009 ± 0.009 control vs 0.06 ± 0.06 upon LPS exposure), IL‐10 (0.006 ± 0.002 control vs 0.013 ± 0.001 upon LPS exposure), and IL‐13 (0.367 ± 0.163 control vs 0.512 ± 0.180 upon LPS exposure) are the proinflammatory CKs that are secreted when STR‐Ms are in NM supplemented with CK (n = 3 donors; Figure 3M). To further explore if cells could retain functionality after grafting in coculture with human brain tissue, presorted STR‐MP cells were labeled with a red tracking dye and grafted onto human cortical slices from resected glioblastoma tissue. At 1 week of coculture, STR‐MP derivative cells showed retained phagocytic abilities when challenged for phagocytic abilities by adding GFP^+^ latex beads in the medium (Figure 3N).

### 3.5 STR‐M in a human ex vivo brain model

**FIGURE 4:**
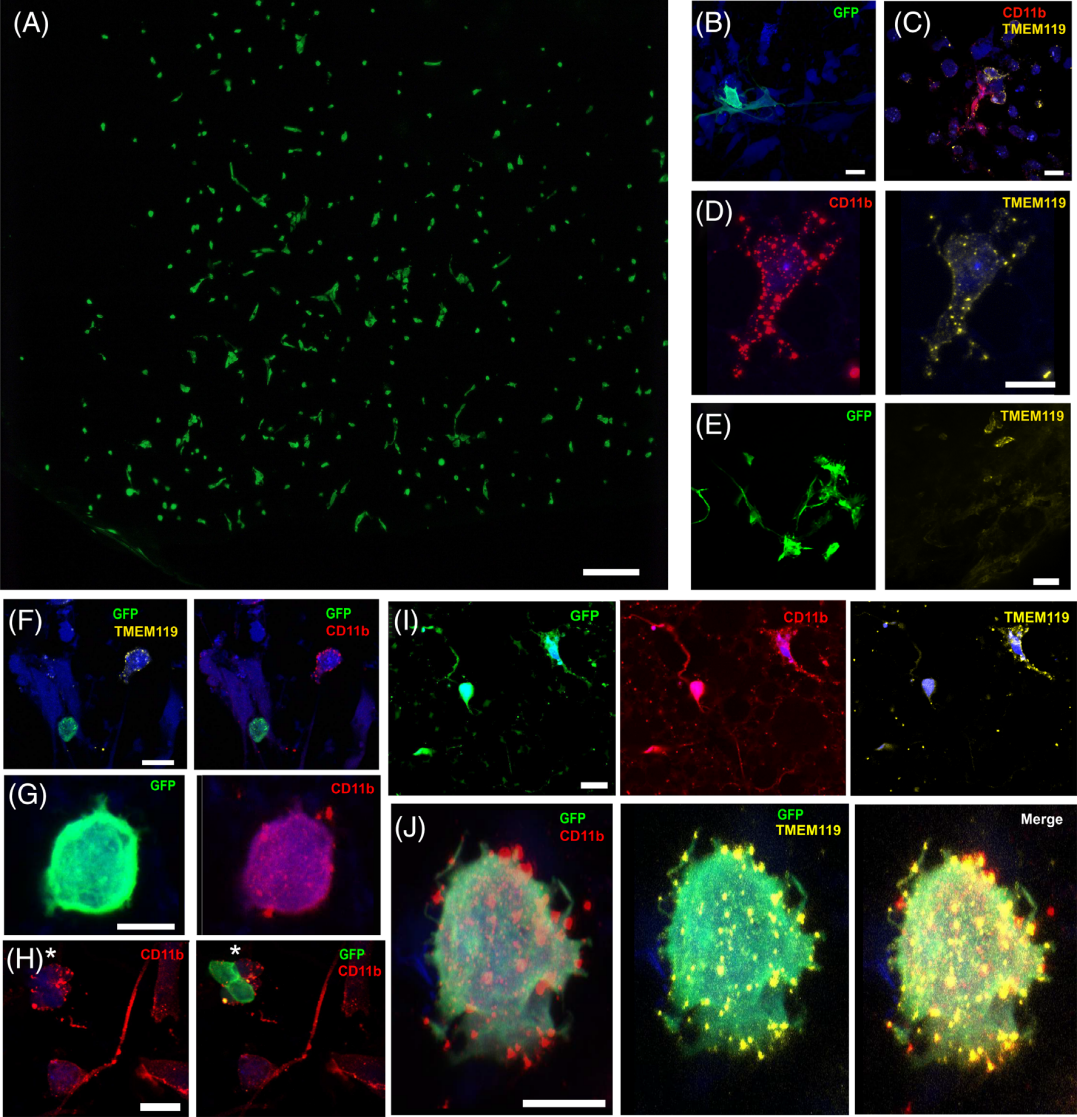
Stromal cells grow in a human brain slice containing glioblastoma. A, Representative image showing GFP^+^ stromal cells after 2‐week coculture with a human brain slice containing glioblastoma. Scale bar, 200 μm. B, Magnification of GFP^+^ stromal cells showing typical morphologies found in organotypic cultures reflecting a more ameboid morphology in contraposition to a more elongated and complex cell. Scale bar, 20 μm. C, Host human tissue ameboid cells showing reactivity to either CD11b or TMEM119. Scale bar, 20 μm. D, Host cells showing microglial ramified morphology and reactivity to CD11b and TMEM119. Scale bar, 20 μm. E, Picture showing elongated morphologies of transplanted GFP^+^ human bone marrow‐derived mesenchymal stromal cells that are negative for TMEM119 immunoreactivity. Scale bar, 50 μm. F, Image showing host microglia immunoreactive to both CD11b and TMEM119 next to an engrafted nonelongated GFP^+^ cell, negative for the same markers. Scale bar, 20 μm. G, GFP^+^ cells with an ameboid shape expressing CD11b but not TMEM119. Scale bar, 10 μm. H, GFP^+^ CD11b^+^ TMEM119^−^ cells that are part of cell niches sharing reactivities toward CD11b. Scale bar, 20 μm. I, Different morphologies are observed for cocultured GFP^+^ cells with the human brain organotypic slice. The asterisk denotes a cell starting to extend ramifications; the arrow denotes a cell with higher reactivity to TMEM119. Scale bar, 20 μm. J, Morphology of a GFP^+^ engrafted cell reactive to CD11b and TMEM119 suggestive of polarization. Scale bar, 10 μm. CD, cluster of differentiation; GFP, green fluorescent protein; TMEM119, transmembrane protein 119

To determine if derivatives of the STR‐MP CD11b^+^ cells can integrate and survive in a human brain environment, GFP^+^ stromal cultures consisting of, on average, 0.236% ± 0.109% GFP^+^ nonhematopoietic CD11b^+^ cells were grafted into ex vivo human brain tissue containing glioblastoma. The ex vivo model was obtained from glioblastoma resections sliced for organotypic culturing. Human brain slices engrafted with the GFP^+^ cells were kept in culture for 2 weeks; slices were fixed and treated for iDISCO immunostaining to identify CD11b and TMEM119 immunoreactive cells among the engrafted GFP^+^ cells. The two cell morphologies—ameboid like and those with soma projections—found in our GFP^+^ engrafted cells were also seen in the slices (Figure 4A,B). In the human tissue, host cells that were either CD11b immunoreactive or TMEM119 immunoreactive were observed (Figure 4C). Likewise, host cells immunoreactive to both markers could be detected, which had a more microglia‐like morphology (Figure 4D). Some of the GFP^+^ engrafted cells with a bone marrow stromal cell‐like morphology showed low immunoreactivity to TMEM119 in comparison with host brain cells (Figure 4E). Some of the ameboid GFP^+^ engrafted cells were not reactive to CD11b^+^, whereas host cells were immunoreactive to both CD11b and TMEM119 (Figure 4F). Some other ameboid GFP^+^ cells were immunoreactive to CD11b (Figure 4G) and even appeared to be integrated into cell groups immunoreactive to CD11b (Figure 4H). CD11b^+^ TMEM119^+^ GFP^+^ cells were also observed (Figure 4I), with morphologies that varied from ameboid‐like to more elaborated morphologies, including cell projections resembling arborizations (Figure 4H,I). Finally, CD11b^+^ TMEM119^+^ GFP^+^ cells morphologies include morphologies similar to polarized macrophages, with radial extensions sprouting from their somas (Figure 4J). Thus, the presence of CD11b^+^ TMEM119^+^ GFP^+^ small cells indicates that STR‐M can survive well in human brain, retain the expression of markers like TMEM119, only expressed in host microglia and not in microglia derived from macrophages, and show morphology polarization in a glioblastoma tumor environment.

## 4 DISCUSSION

### 4.1 A microglia‐like progenitor cell in bone marrow

In the present work we have detected, isolated, cultured, and characterized a novel source of progenitor cells from the hBM that highly resembles a microglial progenitor. Our strategy is based on the analysis of fresh bone marrow extractions from young healthy donors using well‐accepted markers for hematopoietic and mesenchymal bone marrow fractions. In our experimental design, hematopoietic progenitor lineage cells and hematopoietic or mature effector cells are excluded, and all potential myeloid/microglia‐like progenitor cells are detected by medium‐to‐low expression of CD45 and the expression of CD11b.^28^, ^29^ Single‐cell gene expression analysis of this compartment revealed expression of myeloid‐associated genes for microglial cell development, including *RUNX1*, *PU.1*, and *CSF1R*,^1^, ^30^ and human‐specific adult microglial genes, including *P2RY12*, *GPR34*, *C1QA*, and *PROS1*, which are not expressed by human blood‐derived monocytes.^31^ Other canonical microglial genes expressed by the STR‐M progenitor cell compartment include *CD33*, *GPR56*, *CABLES1*, *BHLHE41*, *TMEM119*, *TGFBR1*, *ENTPD1*, *ITGB2*, *ITGAM*, *AIF*, *IRF8*, *ADORA3*, and *PPARD*.^30^, ^31^, ^33^, ^34^, ^35^ We also detected expression of *TREM2* and *CD33*, which are involved in neurodegenerative diseases like Alzheimer's disease and are only expressed by resident microglia.^4^ The unbiased analysis of the single‐cell gene expression panel of the STR‐M progenitor cell compartment indicates that this may include different myeloid cell types. Given that CD11b^+^ cells are CD34^−^, and no Myb‐ and Batf3‐expressing cells were observed, the analysis indicates that a Myb‐independent yolk sac erythromyeloid progenitor may exist in hBM, as described for microglial progenitors.^31^, ^32^, ^33^, ^34^, ^35^, ^46^ Yet another possibility to explain our finding of a CD45^−^ CD11b^+^ progenitor is trans‐differentiation of non‐EMP stem and progenitor cells such as neural crest‐derived bone marrow precursors.^47^ In addition, the observed expression profiles may reflect a maturational process in the STR‐MP compartment, similar to what has been shown in other work for lung cell development.^48^

The STR‐MP cell compartment can be maintained as part of hBM stromal cell cultures selected on their ability to adhere to plastic. That a microglia‐like cell population is dependent on a cell feeder layer is in accordance with the observation that microglial growth is promoted by astrocyte‐like feeder cells, as well as the notion that resident macrophages are shaped by the local microenvironment.^42^, ^49^ hBM MSCs produce a CK environment, which is needed for microglial development, including the expression of M‐CSF and TGF‐β.^33^, ^50^ Our data are in line with this notion as they also indicate that, in low passage cultures, the STR‐MP cell compartment is sensitive to the CK condition. Likewise, we observe that serum‐free conditions substantially decrease hBM MSC numbers in low passage cultures, which ultimately affects the detection of CD11b^+^ cells.

Moreover, STR‐Ms, the STR‐MP derivative defined as CD11b^+^ Iba1^+^ cells, can survive in serum‐free conditions in high passage cultures. In this case, the use of NT in serum‐free medium promotes the appearance of more complex cell morphologies, in accordance with the idea that tropomyosin kinase (Trk) receptors are expressed by microglial cells^51^ and would be supportive of a role for Trk receptors in morphological maturation, as described for both neurons^52^ and astrocytes.^53^

STR‐Ms and STR‐MPs not only can retain classic microglial markers upon culture expansion but also show classic microglial functions like phagocytic abilities and, importantly, M1‐skewed microglial activation upon LPS exposure.^41^ Finally, STR‐Ms survive in human brain tissue and show morphological indication of functional polarization in brain tumor environment. In summary, the hBM STR‐MP compartment can be culture‐expanded and derive STR‐Ms that highly resemble resident microglia at a morphological, genetic, and functional level.

### 4.2 STR‐MP for use in rapid microglia derivative protocols

Our alternative method can derive a mature functional microglia‐like cell type that we propose to call *STR‐M*, to distinguish it from resident microglia, within 1 week from a previously sorted STR‐MP pure cell population and as an alternative to human iPSCs. In vitro, the STR‐M are characterized by microglial marker expression, with ramified to ameboid microglia‐like morphologies, and phagocytic and polarization abilities. In addition, STR‐M survive in human brain tissue, which opens the possibility of their use in clinical translational approaches involving cell‐based therapies.^35^

Current derivative protocols using pluripotent cells depend on the previous generation of human iPSCs, which can greatly delay the derivative process, and first derive hematopoietic‐like progenitors before deriving the microglia‐like cell.^54^ Compared with human iPSC‐derived microglia‐like cells, the present method is faster and eliminates the risk for teratoma formation from implanted iPSC‐derived cells. Moreover, our current protocol skips the need to generate a hematopoietic‐like progenitor and reduces the time for deriving functional microglia‐like cells to 1 week. Other alternatives to work with microglia‐like cells and MSCs so far include the use of murine cell lines like the one described by Rahmat et al as an immortalized HLA‐DR^+^ and HLA‐ABC^+^ BV2 microglial cell line in coculture with MSCs^55^ or the use of CD11b^+^/CD45^−^ enriched microglial cells from adult mouse brain^56^ . It is also possible to differentiate other cells toward microglia‐like cells, such as disclosed by Hinze and Stolzing,^13^ where hBM cells are differentiated toward CD11b^+^/CD45^+^ microglia‐like cells. Nevertheless, the importance of using a CD45^−^ nonhematopoietic progenitor in cell‐based therapies using microglia‐like cells is manifested by the different outcomes that microglia‐like cells derived from hematopoietic lineages can generate during disease compared with host microglia. Hence, the use of a cell closer to human naïve microglia could result in a reliable alternative for treating affections where monocytic and microglial gene lineages show distinct activation patterns and implications in the curse of the disease, that is, neurodegenerative disorders.^14^, ^15^

Lastly, because specific microglial genes are expressed by the STR‐MP, the STR‐MP can potentially be used as a tool for developing diagnostic approaches and in the search for biomarkers. Specific microglial genes are associated with the risk for developing neurodegenerative and psychiatric disorders^4^; thus STR‐MP could be developed as a future diagnostic tool for CNS disorders.

## author contributions

A.B.: experimental design, collection and/or assembly of data, data analysis and interpretation, final approval of manuscript; I.H., A.B.‐S., and A.‐G.H.: collection and/or assembly of data, data analysis and interpretation, final approval of manuscript; A.T.: collection and/or assembly of data, final approval of manuscript; T.D.: data analysis, final approval of manuscript; J.B.: design, provision of study material or patients, final approval of manuscript; T.R.‐M.: conception and design, collection and/or assembly of data, data analysis and interpretation, manuscript writing, final approval of manuscript.

## conflict of interests

T.R.‐M. declared employment and intellectual property rights with Lund University. The other authors declared no potential conflicts of interest. The methodology here described is currently under a patent process (P5297EP00‐CLI).

## ETHICAL STATEMENT

All procedures performed were in accordance with the ethical standards of the institutional and/or national research committee. The ethical permit for hBM extraction was under permit 2009/12 and for use of human brain tissue resections, 212/2007.

## Supporting information


**Data S1**. Supporting information.

**Figure S1**A. Graph representing the presence of STR‐MP cells upon cell culturing. STR‐MP cells, these being represented by the surface marker profile of HLA‐DR‐/CD14‐/CD19‐/CD34‐/CD45‐/CD11b+ represented a 1.23±0.49 % at p6 and 2.55±2.25 % at p11 (one‐tailed unpaired t Test, p^3^0.05; 2 donors, n=4 repetitions). B. Scheme depicting the experimental design for studying non‐hematopoietic CD11b+ cell morphology and culture conditions. Half the fresh bone marrow stromal fraction was stored at ‐180oC in freezing medium. The other half was expanded and infected with lentiviruses expressing GFP and a puromycin resistance gene. Infected cells were treated for 5 days with puromycin and surviving GFP+ stromal cells were used as the feeder cell layer for the sorted non‐hematopoietic CD11b+ cells (n=2 human donors). C. Scheme depicting the experimental design of the in vitro work. After a week of acclimation to the serum‐free media in flasks, cells are seeded on polyornithine and laminin coated coverslips. Cells are then exposed to the NT or CK for 2 or 4 weeks, making short‐ and long‐term timepoints of 3‐ and 5‐weeks total. At these time points, cells can be subjected to functional analysis and/or be fixed with paraformaldehyde 4% (4% PFA) for a posterior immuno‐ and morphological analysis. This analysis was done using Cellomics high content screening, as described in the section for Analysis under Material and Methods. D. Small CD11b+ Iba1+ cells were easily tracked in hBM‐MSCs cultures based on nuclear size and immunoreactivity to Iba1. In brief, cells whose nuclei area was below 400 pixelsÚ2 (corresponding to a nucleus diameter of 10‐20mm) were significantly smaller than the nuclei corresponding to the hBM‐MSCs at the same passage number, p6 (based on the cell characterization of hBM‐MSCs' cultures refs (Ge et al., 2014; Heathman et al., 2015; Torres‐Platas et al., 2014)). High immunoreactivity towards Iba1 is often observed in the small nuclei group across the different culturing conditions. Under the different conditions, the measured sizes were 192.7±13.53 pixel2 vs 1014±181.7 pixel2, BM condition; 193.66±7.79 pixel2 vs 779±65.57 pixel2, BM+NT condition and 179.25±5.43 pixel2 vs 720.25±111.26 pixel2, BM+CK condition; two‐tailed unpaired t Test, *p£0.05; **p£0.005; ***p£0.001 respectively; 2 donors, n=4 repetitions. E. Representative case showing the morphology of human stromal cells at low passages (p<6). Scale bar: 100mm. F. Representative phase contrast image showing high passageculture human stromal cultures associated morphologies (p>6) (Yang et al., 2018). Scale bar: 50mm.

**Figure S2** A. Scheme depicting the microglia phagocytosis experimental design. In brief, preopsonised latex GFP beads are added to the medium where cells are left for 1h at 37oC in the incubator. Cells are then fixed with ice cold 4% PFA solution and analyzed for their reactivity to CD11b and Iba1. B. Scheme depicting the LPS induced activation experimental design. In brief, LPS particles are added to the medium for 24h at 37oC in the incubator. C. Scheme depicting the GFP+ stromal cells co‐culture with human brain tissue containing glioblastoma. After a pre‐differentiating step, on day 7, cells are engrafted onto the organotypic slice on day 7 of a preconditioning step in serum‐free medium and allowed to survive for 2 weeks. The cocultured tissue is then fixed in ice cold 4% PFA for a subsequent iDisco analysis.

**Table S1** Comparative tables showing the % of immunoreactive cells for classical microglia markers in low passage cultures. 1. Table displaying the targeted genes used for the single cell gene analysis. Gene expression by the single cells is denoted by an X. Genes are listed in the same order as listed in Figure 1. In green: genes associated to microglia development; in blue: consensus microglia genes; in yellow: genes associated to maturation; in orange: genes specific to microglia involved neurodegenerative and psychiatric disorders. 2. A. Table showing the calculated area for nuclei as pixelsÚ2 denoting the small and big nuclei present in stroma cultures across the different BM media at 3 and 5 weeks (n=2 donors). B. Table presenting the % of the small cells reactive to CD11b and Iba1 within the small Iba1+ cells compartment and within the total number of cells showed per condition and timepoint under the different BM media (n=2 donors). C. Table presenting the % of the small nuclei immune‐reactive to Iba1 and TMEM119 within the small Iba1+ cell compartment under the different BM conditions (n=2 donors). D. Table showing the % of the small nuclei reactive to Iba1 and CX3CR1 within the population of the small Iba1+ cells under the different BM conditions (n=2 donors). E. Table showing the % of the small cells not reactive to HLA‐DR within the TMEM119 small cells under the different BM conditions (n=2 donors). All data presented as MEAN ± S.E.M. BM: basal or expansion medium (serum‐containing); BM+NT: basal medium supplemented with neurotrophins; BM+CK: basal medium supplemented with cytokines.

**Table S2** Comparative tables showing the % of immunoreactive cells for classical microglia markers in high passage cultures. A. Table showing the % of the cells reactive to CD11b and Iba1 within the Iba1+ cell population under the different BM conditions (n=2 donors). B. Table showing the % of the cells immuno‐reactive to Iba1 and TMEM119 within the population of the small Iba1+ cells under the different BM conditions (n=2 donors). C. Table showing the % cells immune‐reactive to Iba1 and CX3CR1 within the population of the small Iba1+ cells (n=2 donors) in the different BM conditions. D. Table showing the % of the cells no immune‐reactive to HLA‐DR within the TMEM119+ cells under the different BM conditions (n=2 donors). E. Table showing the % of the cells immune‐reactive to CD11b and Iba1 within the population of the small Iba1+ cells under the different serum‐free NM conditions (n=2 donors; *: unpaired, one‐tail Student T test, p<0.05). F. Table showing the % of the cells immune‐reactive to Iba1 and TMEM119 respect of the Iba1+ cells under the different serum‐free NM conditions (n=2 donors). G. Table showing the % of cells immune‐reactive to Iba1 and CX3CR1 respect of the Iba1+ cells under the different serum‐free NM conditions (n=2 donors). H. Table showing the % of the cells not immune‐reactive to HLA‐DR of the TMEM119+ cells under the different serum‐free NM conditions (n=2 donors). All data presented as MEAN ± S.E.M. BM: basal or expansion medium (serum‐containing); BM+NT: basal medium supplemented with neurotrophins; BM+CK: basal medium supplemented with cytokines; NM: neuronal medium (serum free); NM+NT: neuronal medium supplemented with neurotrophins; NM+CK: neuronal medium supplemented with cytokines.

**Table S3** Comparative tables showing the % of different morphologies found in the CD11b+Iba1+ cells in high passage cultures. A. Table showing the % of the ameboid cells immune‐reactive to CD11b and Iba1 within the Iba1+ cell population under the different BM conditions (n=2 donors). B. Table showing the % of the semi‐ramified cells immune‐reactive to CD11b and Iba1 within the Iba1+ cell population under the different BM conditions (n=2 donors). C. Table showing the % of the ramified cells immune‐reactive to CD11b and Iba1 within the Iba1+ cell population under the different BM conditions (n=2 donors). D. Table showing the % of the ameboid cells immune‐reactive to CD11b and Iba1 within the Iba1+ cell population under the different serum‐free NM conditions (n=2 donors). E. Table showing the % of the semi‐ramified cells immune‐reactive to CD11b and Iba1 within the Iba1+ cell population under the different NM conditions (n=2 donors). F. Table showing the % of the ramified cells reactive to CD11b and Iba1 within the Iba1+ cell population under the different NM conditions (n=2 donors). All data presented as MEAN ± S.E.M. BM: basal or expansion medium (serum‐containing); BM+NT: basal medium supplemented with neurotrophins; BM+CK: basal medium supplemented with cytokines; NM: neuronal medium (serum free); NM+NT: neuronal medium supplemented with neurotrophins; M+CK: neuronal medium supplemented with cytokines.

**Table S4** Comparative tables showing the % in the engulfing CD11b+Iba1+ cells in low passage cultures. Percentage of the engulfing CD11b+Iba1+ cells in low passage cultures across the different conditions at 3 (A) and 5 weeks (B). All data presented as MEAN ± S.E.M. (n=2 donors). BM: basal or expansion medium (serum‐containing); BM+NT: basal medium supplemented with neurotrophins; BM+CK: basal medium supplemented with cytokines.

**Table S5** Comparative tables showing the % of the engulfing CD11b+Iba1+ cells in high passage cultures. Percentages of the engulfing CD11b+Iba1+ cells in high passage cultures across the different conditions at 3 (A) and 5 weeks (B). All data presented as MEAN ± S.E.M. (n=2 donors). (C). Percentages of the engulfing CD11b+Iba1+ cells in high passage cultures in 6 days under NM+NT or NM+CK conditions All data presented as MEAN ± S.E.M. (n=3 repetitions). BM: basal or expansion medium (serum‐containing); BM+NT: basal medium supplemented with neurotrophins; BM+CK: basal medium supplemented with cytokines; NM: neuronal medium (serum free); NM+NT: neuronal medium supplemented with neurotrophins; NM+CK: neuronal medium supplemented with cytokines.

**Table S6** A. List showing the primary antibodies that were used in the present work. B. List with the secondary antibodies used in the present work. C. List with the nuclear dyes used in the present work.

## Data Availability

The data that support the findings of this study are available from the corresponding author upon reasonable request.
