# Supplementary material for: The human bone marrow harbors a CD45− CD11B+ cell progenitor permitting rapid microglia‐like cell derivative approaches
Source: Stem Cells Transl Med. 2020 Dec 9;10(4):582–97. doi: 10.1002/sctm.20-0127 (PMC7980218; doi:10.1002/sctm.20-0127)
Supplement: Supplementary file 1 — Data S1. Supporting information. [file SCT3-10-582-s006.docx]

**SUPPLEMENTARY MATERIAL.**

To discard that cultures were not bringing hematopoietic lineages along passages, cells cultures were subjected to immunocytochemistry (ICC) to discard possible expressions of cluster of differentiation 31 and 45 (**CD31, CD45**, respectively; both from Abcam). Cells were studied for these markers under serum-containing and serum-free conditions. Our results show that cell cultures were negative to both CD31 and CD45, confirming the stromal nature of the cell cultures.

To confirm that non-hematopoietic CD11b^+^ cells were negative for the mesenchymal stromal cell marker of cluster of differentiation (**CD90**), cells cultures were subjected to ICC for a possible double expression of cluster of differentiation 90 (CD90; Abcam) and CD11b (CD11b; Abcam). Cells were analyzed for these markers under serum-containing and serum-free conditions. Our results show that cell cultures were negative for CD90.

To discard the possibility of osteoblast lineages expressing CD11b, cultures were subjected to immunostainings for collagen I (Abcam) and osteocalcin (R&D systems) under serum-containing and serum-free conditions. Our results show that the non-hematopoietic CD11b^+^ cell does not express collagen I or osteocalcin under present culturing conditions. The expression of CD33 in some TMEM119 cells clearly supports a myeloid nature of the non-hematopoietic CD11b^+^ cells present in the human BM-MSCs cultures, as it is depicted in the single cell gene analysis (present results, **figure 1B**).
