# Supplementary figures and images for: The human bone marrow harbors a CD45− CD11B+ cell progenitor permitting rapid microglia‐like cell derivative approaches
Source: Stem Cells Transl Med. 2020 Dec 9;10(4):582–97. doi: 10.1002/sctm.20-0127 (PMC7980218; doi:10.1002/sctm.20-0127)

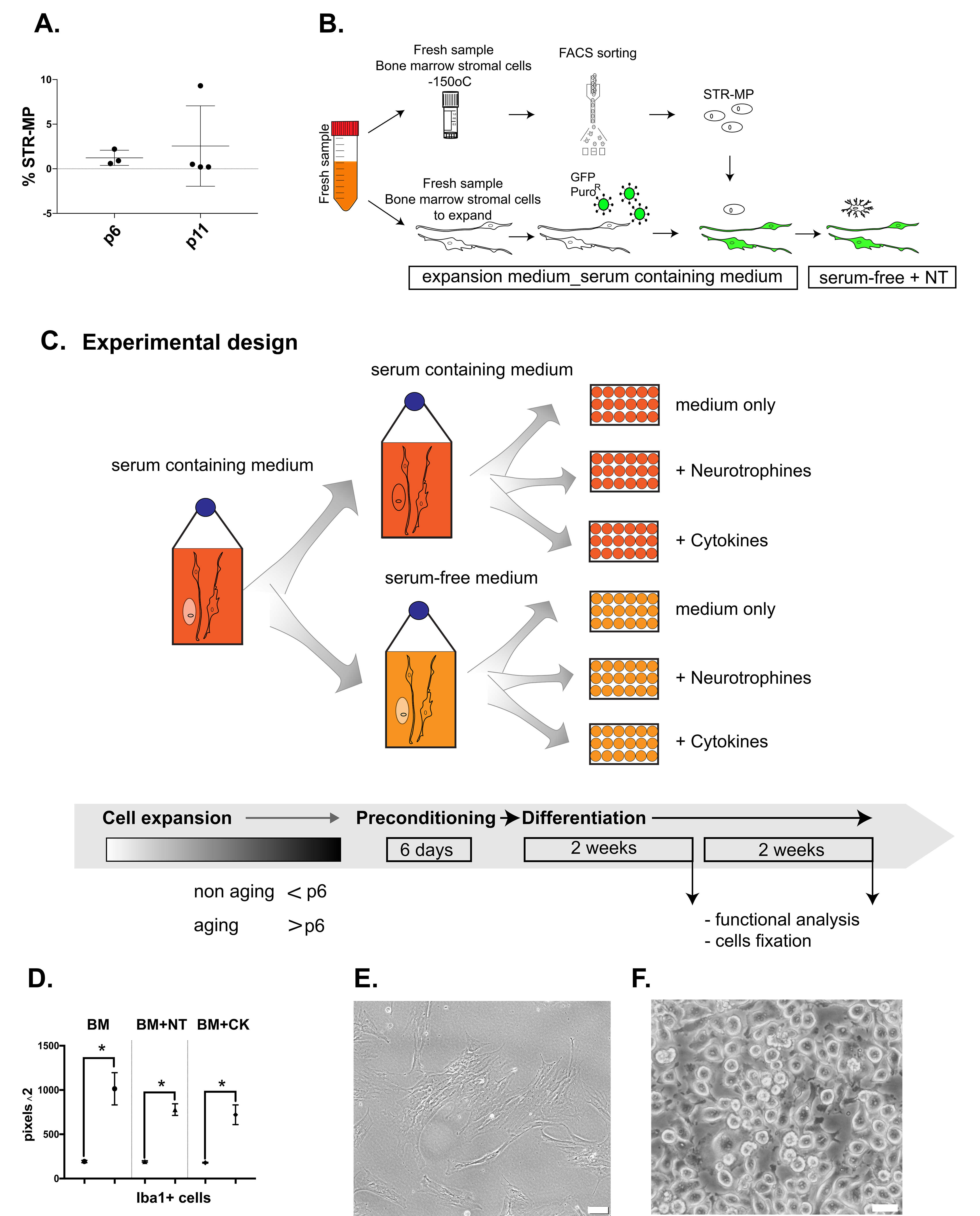

Supplement: Supplementary file 2 — Figure S1A. Graph representing the presence of STR‐MP cells upon cell culturing. STR‐MP cells, these being represented by the surface marker profile of HLA‐DR‐/CD14‐/CD19‐/CD34‐/CD45‐/CD11b+ represented a 1.23±0.49 % at p6 and 2.55±2.25 % at p11 (one‐tailed unpaired t Test, p30.05; 2 donors, n=4 repetitions). B. Scheme depicting the experimental design for studying non‐hematopoietic CD11b+ cell morphology and culture conditions. Half the fresh bone marrow stromal fraction was stored at ‐180oC in freezing medium. The other half was expanded and infected with lentiviruses expressing GFP and a puromycin resistance gene. Infected cells were treated for 5 days with puromycin and surviving GFP+ stromal cells were used as the feeder cell layer for the sorted non‐hematopoietic CD11b+ cells (n=2 human donors). C. Scheme depicting the experimental design of the in vitro work. After a week of acclimation to the serum‐free media in flasks, cells are seeded on polyornithine and laminin coated coverslips. Cells are then exposed to the NT or CK for 2 or 4 weeks, making short‐ and long‐term timepoints of 3‐ and 5‐weeks total. At these time points, cells can be subjected to functional analysis and/or be fixed with paraformaldehyde 4% (4% PFA) for a posterior immuno‐ and morphological analysis. This analysis was done using Cellomics high content screening, as described in the section for Analysis under Material and Methods. D. Small CD11b+ Iba1+ cells were easily tracked in hBM‐MSCs cultures based on nuclear size and immunoreactivity to Iba1. In brief, cells whose nuclei area was below 400 pixelsÚ2 (corresponding to a nucleus diameter of 10‐20mm) were significantly smaller than the nuclei corresponding to the hBM‐MSCs at the same passage number, p6 (based on the cell characterization of hBM‐MSCs' cultures refs (Ge et al., 2014; Heathman et al., 2015; Torres‐Platas et al., 2014)). High immunoreactivity towards Iba1 is often observed in the small nuclei group across the different cultu [file SCT3-10-582-s003.tif]

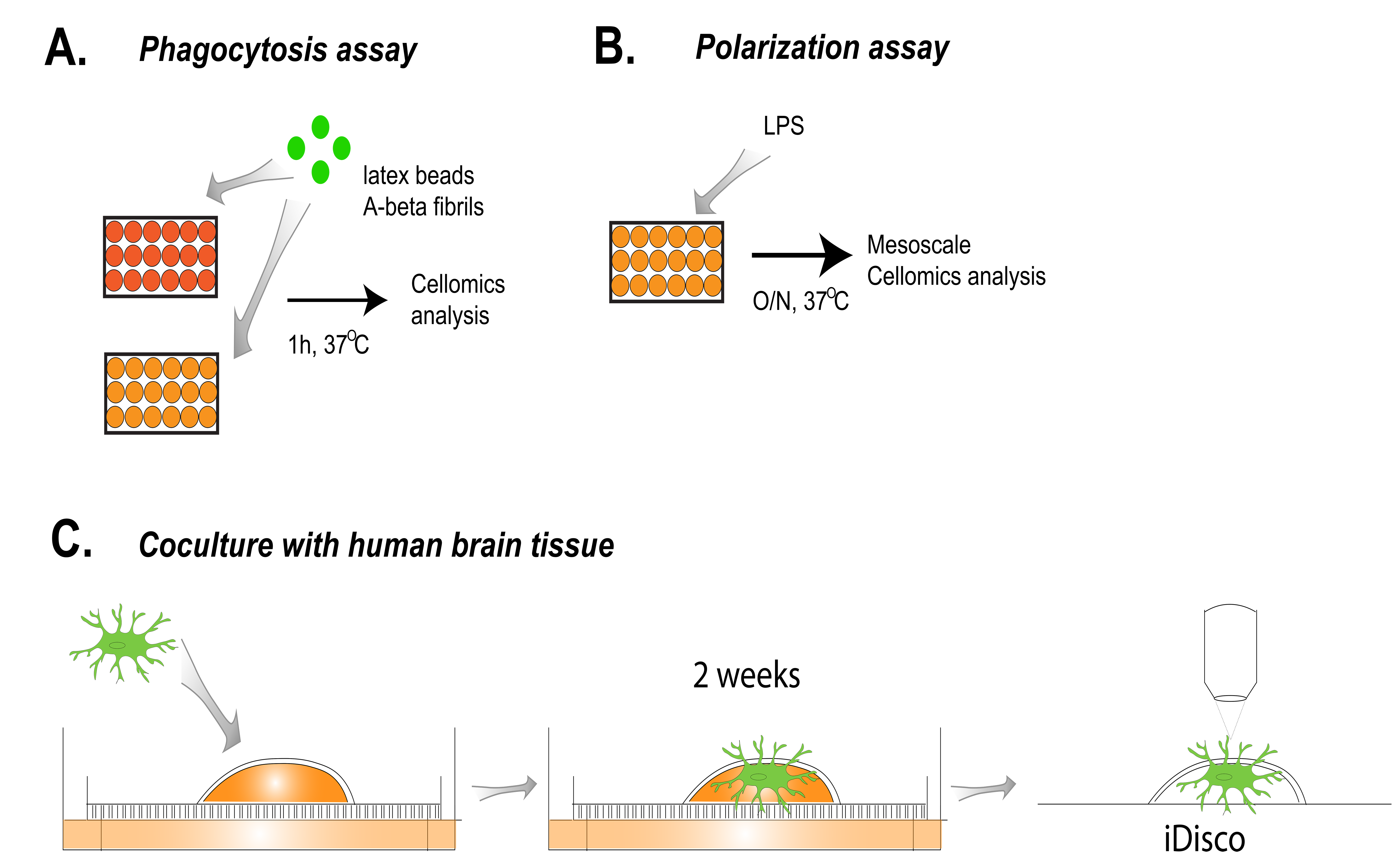

Supplement: Supplementary file 3 — Figure S2 A. Scheme depicting the microglia phagocytosis experimental design. In brief, preopsonised latex GFP beads are added to the medium where cells are left for 1h at 37oC in the incubator. Cells are then fixed with ice cold 4% PFA solution and analyzed for their reactivity to CD11b and Iba1. B. Scheme depicting the LPS induced activation experimental design. In brief, LPS particles are added to the medium for 24h at 37oC in the incubator. C. Scheme depicting the GFP+ stromal cells co‐culture with human brain tissue containing glioblastoma. After a pre‐differentiating step, on day 7, cells are engrafted onto the organotypic slice on day 7 of a preconditioning step in serum‐free medium and allowed to survive for 2 weeks. The cocultured tissue is then fixed in ice cold 4% PFA for a subsequent iDisco analysis. [file SCT3-10-582-s002.tif]
