# Supplementary material for: The human bone marrow harbors a CD45− CD11B+ cell progenitor permitting rapid microglia‐like cell derivative approaches
Source: Stem Cells Transl Med. 2020 Dec 9;10(4):582–97. doi: 10.1002/sctm.20-0127 (PMC7980218; doi:10.1002/sctm.20-0127)
Supplement: Supplementary file 4 — Table S1 Comparative tables showing the % of immunoreactive cells for classical microglia markers in low passage cultures. 1. Table displaying the targeted genes used for the single cell gene analysis. Gene expression by the single cells is denoted by an X. Genes are listed in the same order as listed in Figure 1. In green: genes associated to microglia development; in blue: consensus microglia genes; in yellow: genes associated to maturation; in orange: genes specific to microglia involved neurodegenerative and psychiatric disorders. 2. A. Table showing the calculated area for nuclei as pixelsÚ2 denoting the small and big nuclei present in stroma cultures across the different BM media at 3 and 5 weeks (n=2 donors). B. Table presenting the % of the small cells reactive to CD11b and Iba1 within the small Iba1+ cells compartment and within the total number of cells showed per condition and timepoint under the different BM media (n=2 donors). C. Table presenting the % of the small nuclei immune‐reactive to Iba1 and TMEM119 within the small Iba1+ cell compartment under the different BM conditions (n=2 donors). D. Table showing the % of the small nuclei reactive to Iba1 and CX3CR1 within the population of the small Iba1+ cells under the different BM conditions (n=2 donors). E. Table showing the % of the small cells not reactive to HLA‐DR within the TMEM119 small cells under the different BM conditions (n=2 donors). All data presented as MEAN ± S.E.M. BM: basal or expansion medium (serum‐containing); BM+NT: basal medium supplemented with neurotrophins; BM+CK: basal medium supplemented with cytokines. [file SCT3-10-582-s004.docx]

1.

| **Genes** | Expresssed | **Genes** | Expresssed | **Genes** | Expresssed | **Genes** | Expresssed |
| --- | --- | --- | --- | --- | --- | --- | --- |
| IRF8 | X | TGFB1 | X | ITGAM | X | TREM2 | X |
| PU.1 | X | TGFBR1 | X | ITGB2 | X | CD33 | X |
| RUNX1 | X | P2RY12 | X | CSF1R | X | HEXB | X |
| MYB | X | C1QA | X | ADORA3 | X | DRD4 | X |
| IRF4 | X | PROS1 | X | AIF1 | X | SLC6A3 |  |
| ID2 | X | GAS6 | X | GPR56 | X | CACNA2C |  |
| BATF3 | X | CABLES1 | X | BIN1 | X | MTHFR | X |
| KLF4 | X | BHLHE41 | X | APOE | X |  |  |
| TMEM119 | X | SLCO2B |  |  |  |  |  |
| MERTK1 | X | SLC7A8 | X |  |  |  |  |
| GPR34 | X | PPARD | X |  |  |  |  |
| ENTPD1 | X | OLFML3 | X |  |  |  |  |

2.

| **A.** | Small Iba1+ pixel∧2 | Big Iba1+ pixel∧2 |
| --- | --- | --- |
| 3 weeks  5 weeks | 192.7±13.53 | 1014±181.7 |
|  | 191.64±6.9 | 728.16±39.73 |

| **B.** | %Iba1+CD11b+/Iba1+ small | %Iba1+CD11b+/total small cells | %Iba1+CD11b+/total cells |
| --- | --- | --- | --- |
| BM_3 weeks  BM_5 weeks | 25± 25 | 0.06±0.06 | 0.006±0.006 |
|  | 13.02± 3.6 | 0.55±0.25 | 0.002±0.001 |
| BM+NT_3 weeks  BM+NT_5 weeks | 22.22± 22.22 | 0.07±0.07 | 0.003±0.003 |
|  | 18.08± 8.99 | 3.79±3.63 | 0.012±0.01 |
| BM+CK_3 weeks  BM+CK_5 weeks | 55.55± 44.44 | 0.26±0.12 * | 0.03±0.02 |
|  | 20.90± 6.87 | 0.81±0.09 * | 0.004±0.002 |

| **C.** | %Iba1+TMEM119+/Iba1+ small |
| --- | --- |
| BM_3 weeks  BM_5 weeks | 50± 50 |
|  | 63.33±3.33 |
| BM+NT_3 weeks  BM+NT_5 weeks | 58.33±8.33 |
|  | 66.7±10.7 |
| BM+CK_3 weeks  BM+CK_5 weeks | 58.33±8.33 |
|  | 59.16±34.16 |

| **D.** | % CX3CR1+ Iba1+/Iba1+ small |
| --- | --- |
| BM_3 weeks  BM_5 weeks | 100±0 |
|  | 93.52±1.21 |
| BM+NT_3 weeks  BM+NT_5 weeks | 100±0 |
|  | 96±0 |
| BM+CK_3 weeks  BM+CK_5 weeks | 100±0 |
|  | 98.91±1.08 |

| **E.** | %HLA DR- TMEM119+/TMEM119+ small |
| --- | --- |
| BM_3 weeks  BM_5 weeks | 83.33±16.66 |
|  | 100±0 |
| BM+NT_3 weeks  BM+NT_5 weeks | 100±0 |
|  | 93.75±6.25 |
| BM+CK_3 weeks  BM+CK_5 weeks | 100±0 |
|  | 87.5±12.5 |

All data presented as MEAN ± S.E.M.
