# Supplementary material for: The human bone marrow harbors a CD45− CD11B+ cell progenitor permitting rapid microglia‐like cell derivative approaches
Source: Stem Cells Transl Med. 2020 Dec 9;10(4):582–97. doi: 10.1002/sctm.20-0127 (PMC7980218; doi:10.1002/sctm.20-0127)
Supplement: Supplementary file 5 — Table S2 Comparative tables showing the % of immunoreactive cells for classical microglia markers in high passage cultures. A. Table showing the % of the cells reactive to CD11b and Iba1 within the Iba1+ cell population under the different BM conditions (n=2 donors). B. Table showing the % of the cells immuno‐reactive to Iba1 and TMEM119 within the population of the small Iba1+ cells under the different BM conditions (n=2 donors). C. Table showing the % cells immune‐reactive to Iba1 and CX3CR1 within the population of the small Iba1+ cells (n=2 donors) in the different BM conditions. D. Table showing the % of the cells no immune‐reactive to HLA‐DR within the TMEM119+ cells under the different BM conditions (n=2 donors). E. Table showing the % of the cells immune‐reactive to CD11b and Iba1 within the population of the small Iba1+ cells under the different serum‐free NM conditions (n=2 donors; *: unpaired, one‐tail Student T test, p<0.05). F. Table showing the % of the cells immune‐reactive to Iba1 and TMEM119 respect of the Iba1+ cells under the different serum‐free NM conditions (n=2 donors). G. Table showing the % of cells immune‐reactive to Iba1 and CX3CR1 respect of the Iba1+ cells under the different serum‐free NM conditions (n=2 donors). H. Table showing the % of the cells not immune‐reactive to HLA‐DR of the TMEM119+ cells under the different serum‐free NM conditions (n=2 donors). All data presented as MEAN ± S.E.M. BM: basal or expansion medium (serum‐containing); BM+NT: basal medium supplemented with neurotrophins; BM+CK: basal medium supplemented with cytokines; NM: neuronal medium (serum free); NM+NT: neuronal medium supplemented with neurotrophins; NM+CK: neuronal medium supplemented with cytokines. [file SCT3-10-582-s007.docx]

| **A.** | %Iba1+CD11b+/Iba1+ cells |
| --- | --- |
| BM_3 weeks  BM_5 weeks | 20.79 ± 18.86 |
|  | 0.17 ± 0.15 |
| BM+NT_3 weeks  BM+NT_5 weeks | 46.96 ± 11.15 |
|  | 1.66 ± 1.65 |
| BM+CK_3 weeks  BM+CK_5 weeks | 8.42 ± 6.63 |
|  | 1.86 ± 0.96 |

| **E.** | %Iba1+CD11b+/Iba1+ cells |
| --- | --- |
| NM_3 weeks  NM_5 weeks | 23.55 ± 6.88 |
|  | 2.72 ± 2.4 |
| NM+NT_3 weeks  NM+NT_5 weeks | 23.07 ± 1.64 |
|  | 0.13 ± 0.02 |
| NM+CK_3 weeks  NM+CK_5 weeks | 24.22 ± 24.21 |
|  | 0.01 ± 0.008 |

| **B.** | %Iba1+TMEM119+/Iba1+ cells |
| --- | --- |
| BM_3 weeks  BM_5 weeks | 34.06 ± 24.31 |
|  | 4.16 ± 4.14 |
| BM+NT_3 weeks  BM+NT_5 weeks | 74.15 ± 4.61 |
|  | 0.19 ± 0.15 |
| BM+CK_3 weeks  BM+CK_5 weeks | 44.53 ± 26.34 |
|  | 0.04 ± 0.04 |

| **F.** | %Iba1+TMEM119+/Iba1+ cells |
| --- | --- |
| NM_3 weeks  NM_5 weeks | 51.52 ± 22.94 |
|  | 5.03 ± 4.87 |
| NM+NT_3 weeks  NM+NT_5 weeks | 74.55 ± 5.13 |
|  | 2.29 ± 2.25 |
| NM+CK_3 weeks  NM+CK_5 weeks | 86.67 ± 2.21 |
|  | 45.55 ± 45.01 |

| **C.** | % CX3CR1+ Iba1+/Iba1+ cells |
| --- | --- |
| BM_3 weeks  BM_5 weeks | 100 ± 0 |
|  | 100 ± 0 |
| BM+NT_3 weeks  BM+NT_5 weeks | 100 ± 0 |
|  | 100 ± 0 |
| BM+CK_3 weeks  BM+CK_5 weeks | 100 ± 0 |
|  | 100 ± 0 |

| **G.** | % CX3CR1+ Iba1+/Iba1+ cells |
| --- | --- |
| NM_3 weeks  NM_5 weeks | 100 ± 0 |
|  | 100 ± 0 |
| NM+NT_3 weeks  NM+NT_5 weeks | 100 ± 0 |
|  | 100 ± 0 |
| NM+CK_3 weeks  NM+CK_5 weeks | 100 ± 0 |
|  | 100 ± 0 |

| **D.** | %HLA DR- TMEM119+/TMEM119+ cells |
| --- | --- |
| BM_3 weeks  BM_5 weeks | 94.35 ± 5.6 |
|  | 99.79 ± 0.2 |
| BM+NT_3 weeks  BM+NT_5 weeks | 80.85 ± 19.14 |
|  | 98.82 ± 1.17 |
| BM+CK_3 weeks  BM+CK_5 weeks | 94.13 ± 5.8 |
|  | 100 ± 0 |

| **H.** | %HLA DR- TMEM119+/TMEM119+ cells |
| --- | --- |
| NM_3 weeks  NM_5 weeks | 99.93 ± 0.07 |
|  | 99.79 ± 0.13 |
| NM+NT_3 weeks  NM+NT_5 weeks | 99.04 ± 0.96 |
|  | 98.02 ± 1.94 |
| NM+CK_3 weeks  NM+CK_5 weeks | 100 ± 0 |
|  | 99.99 ± 0.006 |

All data presented as MEAN ± S.E.M.
