# Supplementary material for: The human bone marrow harbors a CD45− CD11B+ cell progenitor permitting rapid microglia‐like cell derivative approaches
Source: Stem Cells Transl Med. 2020 Dec 9;10(4):582–97. doi: 10.1002/sctm.20-0127 (PMC7980218; doi:10.1002/sctm.20-0127)
Supplement: Supplementary file 6 — Table S3 Comparative tables showing the % of different morphologies found in the CD11b+Iba1+ cells in high passage cultures. A. Table showing the % of the ameboid cells immune‐reactive to CD11b and Iba1 within the Iba1+ cell population under the different BM conditions (n=2 donors). B. Table showing the % of the semi‐ramified cells immune‐reactive to CD11b and Iba1 within the Iba1+ cell population under the different BM conditions (n=2 donors). C. Table showing the % of the ramified cells immune‐reactive to CD11b and Iba1 within the Iba1+ cell population under the different BM conditions (n=2 donors). D. Table showing the % of the ameboid cells immune‐reactive to CD11b and Iba1 within the Iba1+ cell population under the different serum‐free NM conditions (n=2 donors). E. Table showing the % of the semi‐ramified cells immune‐reactive to CD11b and Iba1 within the Iba1+ cell population under the different NM conditions (n=2 donors). F. Table showing the % of the ramified cells reactive to CD11b and Iba1 within the Iba1+ cell population under the different NM conditions (n=2 donors). All data presented as MEAN ± S.E.M. BM: basal or expansion medium (serum‐containing); BM+NT: basal medium supplemented with neurotrophins; BM+CK: basal medium supplemented with cytokines; NM: neuronal medium (serum free); NM+NT: neuronal medium supplemented with neurotrophins; M+CK: neuronal medium supplemented with cytokines. [file SCT3-10-582-s009.docx]

| **A**. Ameboid | %Iba1+CD11b+/Iba1+ cells |
| --- | --- |
| BM_3 weeks  BM_5 weeks | 6.23 ± 1.65 |
|  | 49.91 ± 7.68 |
| BM+NT_3 weeks  BM+NT_5 weeks | 14.02 ± 1.35 |
|  | 41.15 ± 7.03 |
| BM+CK_3 weeks  BM+CK_5 weeks | 17.68 ± 1.77 |
|  | 29.74 ± 6.12 |

| **D.** ameboid | %Iba1+CD11b+/Iba1+ cells |
| --- | --- |
| NM_3 weeks  NM_5 weeks | 13.64 ± 3.25 |
|  | 16.76 ± 4.75 |
| NM+NT_3 weeks  NM+NT_5 weeks | 35.88 ± 4.21 |
|  | 17.18 ± 2.28 |
| NM+CK_3 weeks  NM+CK_5 weeks | 39.03 ± 7.39 |
|  | 21.35 ± 4.89 |

| **B**.Semi-ramified | %Iba1+CD11b+/Iba1+ cells |
| --- | --- |
| BM_3 weeks  BM_5 weeks | 0.46 ± 0.22 |
|  | 6.28 ± 1.61 |
| BM+NT_3 weeks  BM+NT_5 weeks | 1.46 ± 0.43 |
|  | 3.32 ± 1.19 |
| BM+CK_3 weeks  BM+CK_5 weeks | 5.25 ± 1.23 |
|  | 7.78 ± 2.32 |

| **E.** Semi-ramified | %Iba1+CD11b+/Iba1+ cells |
| --- | --- |
| NM_3 weeks  NM_5 weeks | 8,34 ± 2.93 |
|  | 14.04 ± 2.98 |
| NM+NT_3 weeks  NM+NT_5 weeks | 19.2 ± 3.63 |
|  | 16.55 ± 2.64 |
| NM+CK_3 weeks  NM+CK_5 weeks | 10.37 ± 3.37 |
|  | 21.07 ± 5.92 |

| **C.** Ramified | %Iba1+CD11b+/Iba1+ cells |
| --- | --- |
| BM_3 weeks  BM_5 weeks | 0.02 ± 0.02 |
|  | 1.83 ± 1.08 |
| BM+NT_3 weeks  BM+NT_5 weeks | 0.11 ± 0.06 |
|  | 0.79 ± 0.55 |
| BM+CK_3 weeks  BM+CK_5 weeks | 0.047 ± 0.02 |
|  | 7.6 ± 3.19 |

| **F.** Ramified | %Iba1+CD11b+/Iba1+ cells |
| --- | --- |
| NM_3 weeks  NM_5 weeks | 1.24 ± 0.92 |
|  | 28.43 ± 7.2 |
| NM+NT_3 weeks  NM+NT_5 weeks | 5.34 ± 1.6 |
|  | 24.63 ± 5.94 |
| NM+CK_3 weeks  NM+CK_5 weeks | 3.7 ± 3.31 |
|  | 14.24 ± 6.93 |

All data presented as MEAN ± S.E.M.
