# Supplementary material for: The human bone marrow harbors a CD45− CD11B+ cell progenitor permitting rapid microglia‐like cell derivative approaches
Source: Stem Cells Transl Med. 2020 Dec 9;10(4):582–97. doi: 10.1002/sctm.20-0127 (PMC7980218; doi:10.1002/sctm.20-0127)
Supplement: Supplementary file 7 — Table S4 Comparative tables showing the % in the engulfing CD11b+Iba1+ cells in low passage cultures. Percentage of the engulfing CD11b+Iba1+ cells in low passage cultures across the different conditions at 3 (A) and 5 weeks (B). All data presented as MEAN ± S.E.M. (n=2 donors). BM: basal or expansion medium (serum‐containing); BM+NT: basal medium supplemented with neurotrophins; BM+CK: basal medium supplemented with cytokines. [file SCT3-10-582-s005.docx]

| **A. 3 weeks** | **% engulfing Iba1+ CD11b+ cells** | **nr. beads/ Iba1+ CD11b+ cells** |
| --- | --- | --- |
| BM BM+NT  BM+CK | 0 ± 0 | 0 ± 0 |
|  | 0 ± 0 | 0 ± 0 |
|  | 0 ± 0 | 0 ± 0 |

| **B. 5 weeks** | **% engulfing Iba1+ CD11b+ cells** | **nr. beads/ Iba1+ CD11b+ cells** |
| --- | --- | --- |
| BM BM+NT  BM+CK | 16.67 ± 16.67 | 0.5 ± 0.5 |
|  | 0 ± 0 | 0 ± 0 |
|  | 12.5 ± 12.5 | 1 ± 1 |

All data presented as MEAN ± S.E.M.
