# Supplementary material for: The human bone marrow harbors a CD45− CD11B+ cell progenitor permitting rapid microglia‐like cell derivative approaches
Source: Stem Cells Transl Med. 2020 Dec 9;10(4):582–97. doi: 10.1002/sctm.20-0127 (PMC7980218; doi:10.1002/sctm.20-0127)
Supplement: Supplementary file 8 — Table S5 Comparative tables showing the % of the engulfing CD11b+Iba1+ cells in high passage cultures. Percentages of the engulfing CD11b+Iba1+ cells in high passage cultures across the different conditions at 3 (A) and 5 weeks (B). All data presented as MEAN ± S.E.M. (n=2 donors). (C). Percentages of the engulfing CD11b+Iba1+ cells in high passage cultures in 6 days under NM+NT or NM+CK conditions All data presented as MEAN ± S.E.M. (n=3 repetitions). BM: basal or expansion medium (serum‐containing); BM+NT: basal medium supplemented with neurotrophins; BM+CK: basal medium supplemented with cytokines; NM: neuronal medium (serum free); NM+NT: neuronal medium supplemented with neurotrophins; NM+CK: neuronal medium supplemented with cytokines. [file SCT3-10-582-s001.docx]

| **A. 3 weeks** | **% engulfing Iba1+ CD11b+ cells** | **nr. beads/ Iba1+ CD11b+ cells** | **nr. beads/ TMEM119+ HLA-DR+ cells** |
| --- | --- | --- | --- |
| BM BM+NT  BM+CK | 2.07 ± 1.18 | 2.51 ± 0.85 | 0.33 ± 0.19 |
|  | 4.71 ± 2.82 | 2.55 ± 0.74 | 0.22 ± 0.05 |
|  | 4.95 ± 4.95 | 0.65 ± 1.41 | 0.31 ± 0.24 |
| NM NM+NT  NM+CK | 50.10 ± 49.89 | 1.10 ± 0.10 | 0.49 ± 0.49 |
|  | 0.26± 0.26 | 0.66 ± 0.66 | 0.04 ± 0.04 |
|  | 0.49 ± 0.49 | 1.04 ± 1.04 | 0.14 ± 0.14 |

| **B. 5 weeks** | **% engulfing Iba1+ CD11b+ cells** | **nr. beads/ Iba1+ CD11b+ cells** | **nr. beads/ TMEM119+ HLA-DR+ cells** |
| --- | --- | --- | --- |
| BM BM+NT  BM+CK | 1.26 ± 1.26 | 2.25 ± 2.25 | 0.15 ± 0.09 |
|  | 1.81 ± 1.81 | 1.2 ± 1.2 | 0.15 ± 0.09 |
|  | 3.64 ± 0.10 | 2.78 ± 1.41 | 0.10 ± 0.005 |
| NM NM+NT  NM+CK | 3.39 ± 0.6 | 2.65 ± 0.65 | 1.1 ± 1.08 |
|  | 3.7 ± 3.7 | 4.25 ± 4.25 | 0.26 ± 0.24 |
|  | 0 ± 0 | 0 ± 0 | 0.04 ± 0.04 |

| **C. 1 week** | **% Aβ engulfing Iba1+ CD11b+ cells** |
| --- | --- |
| NM+NT  NM+CK | 9.69 ± 1.44 |
|  | 57.34 ± 23.05 |

All data presented as MEAN ± S.E.M.
