# Supplementary material for: The human bone marrow harbors a CD45− CD11B+ cell progenitor permitting rapid microglia‐like cell derivative approaches
Source: Stem Cells Transl Med. 2020 Dec 9;10(4):582–97. doi: 10.1002/sctm.20-0127 (PMC7980218; doi:10.1002/sctm.20-0127)
Supplement: Supplementary file 9 — Table S6 A. List showing the primary antibodies that were used in the present work. B. List with the secondary antibodies used in the present work. C. List with the nuclear dyes used in the present work. [file SCT3-10-582-s008.docx]

1. **Primary antibodies**

| **Host** | **Target** | **Commercial house** | **Reference number** | **Dilution** |
| --- | --- | --- | --- | --- |
| Rabbit | CD11b | Abcam | ab133357 | 1 in 200 |
| Goat | Iba1 | Abcam | ab5076 | 1 in 200 |
| Rabbit | TMEM119 | Abcam | ab185333 | 1 in 200 |
| Mouse | CD33 | Abcam | ab30371 | 1 in 200 |
| Mouse | HLA-DR | Abcam | ab20181 | 1 in 200 |
| Chicken | GFP | Abcam | ab13970 | 1 in 500 |
| Rabbit | CX3CR1 | Abcam | ab185333 | 1 in 200 |
| Mouse | CD11b | BIORAD | MCA711 | 1 in 200 |
| Rabbit | CD45 | Abcam | ab10558 | 1 in 200 |
| Mouse | CD40 | Invitrogen | MA5-15535 | 1 in 200 |
| Rabbit | CD31 | Abcam | ab28364 | 1 in 200 |
| Rabbit | CD31 | Abcam | ab32457 | 1 in 200 |
| Rabbit | CD90 | Abcam | ab92574 | 1 in 200 |
| Rat | CD45 | Abcam | ab25386 | 1 in 200 |
| Mouse | Anti h/r Osteocalcin | R&D Systems | MAB1419 | 1 in 200 |
| Mouse | Collagen I | Abcam | ab88147 | 1 in 200 |

1. **Secondary antibodies**

| **Host** | **Target** | **Dye** | **Commercial house** | **Reference number** | **Dilution** |
| --- | --- | --- | --- | --- | --- |
| Donkey | Goat | Cy3 | Jacksson Immuno Research | 705-165-147 | 1 in 200 |
| Donkey | Mouse | Cy3 | Jacksson Immuno Research | 715-165-150 | 1 in 200 |
| Donkey | Rabbit | 647 | Jacksson Immuno Research | 711-605-152 | 1 in 200 |
| Donkey | Chicken | 488 | Jacksson Immuno Research | 703-545-155 | 1 in 200 |

| **C.** | Nuclear Stainings | **Commercial house** | **Reference number** |
| --- | --- | --- | --- |
| iDisco | PO-PRO-1 Iodide | Invitrogen | P3589 |
| ICCs | Prolong Gold Antifade Mountant with DAPI | Thermo Fisher Scientific | P36931 |
